# Cell type- and factor-specific nonsense-mediated RNA decay

**DOI:** 10.1093/nar/gkaf395

**Published:** 2025-05-14

**Authors:** Kun Tan, Jonathan Sebat, Miles F Wilkinson

**Affiliations:** Department of Obstetrics, Gynecology, and Reproductive Sciences, School of Medicine, University of California San Diego, La Jolla, CA 92093, United States; Department of Psychiatry, Department of Cellular and Molecular Medicine, School of Medicine, University of California San Diego, La Jolla, CA 92093, United States; Institute of Genomic Medicine, University of California San Diego, La Jolla, CA 92093, United States; Department of Obstetrics, Gynecology, and Reproductive Sciences, School of Medicine, University of California San Diego, La Jolla, CA 92093, United States; Institute of Genomic Medicine, University of California San Diego, La Jolla, CA 92093, United States

## Abstract

Nonsense-mediated RNA decay (NMD) is a highly conserved RNA turnover pathway that influences several biological processes. Specific features in messenger RNAs (mRNAs) have been found to trigger decay by NMD, leading to the assumption that NMD sensitivity is an intrinsic quality of a given transcript. Here, we provide evidence that, instead, an overriding factor dictating NMD sensitivity is the cell environment. Using several genome-wide techniques to detect NMD-target mRNAs, we find that hundreds of mRNAs are sensitized to NMD as human embryonic stem cells progress to form neural progenitor cells. Another class of mRNAs escape from NMD during this developmental progression. We show that the differential sensitivity to NMD extends to *in vivo* scenarios, and that the RNA-binding protein, HNRNPL, has a role in cell type-specific NMD. We also addressed another issue in the field—whether NMD factors are core or branch-specific in their action. Surprisingly, we found that UPF3B, an NMD factor critical for the nervous system, shares only 30% of NMD-target transcripts with the core NMD factor UPF2. Together, our findings have implications for how NMD is defined and measured, how NMD acts in different biological contexts, and how different NMD branches influence human diseases.

## Graphical Abstract

**Graphical Abstract:**
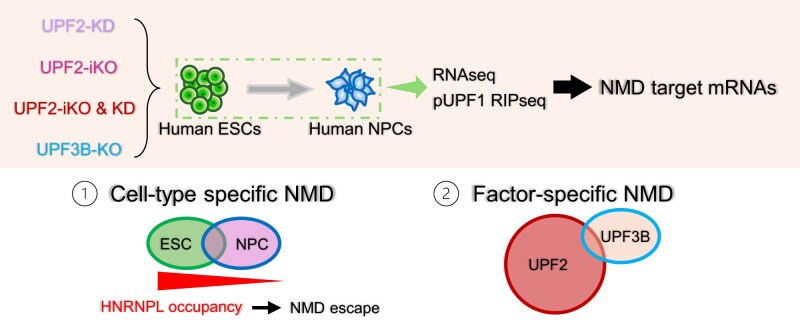

## Introduction

RNA turnover (decay) is a highly regulated process that works together with transcriptional regulation to dictate steady-state messenger RNA (mRNA) levels [1, 2]. While RNA turnover regulation has been shown to have roles in wide spectrum of biological events, the mechanisms underlying RNA turnover regulation are poorly understood, particularly with regard to developmental processes.

Arguably, the best-studied RNA turnover pathway is nonsense-mediated RNA decay (NMD). This highly conserved RNA decay pathway was originally discovered by virtue of its ability to degrade aberrant RNAs harboring premature termination codons. In this role, NMD protects cells from truncated, potentially toxic, dominant-negative proteins [3–6]. Subsequently, it was discovered that NMD degrades many normal RNAs, with loss or disruption of NMD leading to dysregulation of ∼5%–20% of the normal transcriptome in species spanning the phylogenetic scale [7–9].

NMD is well-studied at the biochemical level [3, 4]. UPF1 and UPF2 serve as core factors critical for NMD from yeast to humans. UPF1 is a highly regulated RNA helicase essential for NMD that translocates on RNA substrates when it hydrolyses ATP [4–6]. UPF1 has a helicase domain that is critical for its function in NMD; UPF1 also has regulatory domains, including the SQ domain that is phosphorylated in metazoans by another protein critical for NMD—the SMG1 protein kinase [10, 11]. UPF1 directly binds UPF2 [4], an adaptor protein that triggers UPF1 to undergo a conformational change that activates its RNA-helicase activity [10, 12]. UPF2 also facilitates UPF1’s ATPase activity [11], and it also stimulates phosphorylation of UPF1 by SMG1 [13]. In addition to its UPF1-promoting roles, UPF2 serves as an NMD scaffolding factor through one of its three tandem MIF4G domains that directly bind to other NMD factors, including SMG1 and UPF3B [4–6, 14]. UPF3B is an adaptor protein that binds not only UPF2 but also the exon-junction complex (EJC), an NMD-promoting molecular complex consisting of the trimer core—eIF4AIII, Y14 (RBM8A), and MAGOH—that binds just upstream of exon–exon junctions in RNAs after they are spliced in the nucleus [3]. The EJC travels with spliced mRNAs into the cytoplasm and delivers an NMD-promoting signal after translation termination [15].

The discovery that NMD targets many normal mRNAs for decay raised the possibility that the function of NMD extends beyond quality control. This notion has been strongly supported by studies showing that NMD factors influence many fundamental processes, including development, cell differentiation, cell proliferation, the integrated stress response, and autophagy [3, 4, 16–30]. A biological system particularly impacted by NMD is the nervous system. NMD has been shown to be critical for neural development, neural stem cell differentiation decisions, axon guidance, and synaptic plasticity [18, 19]. In humans, mutations in the NMD gene, *UPF3B*, cause intellectual disability [18, 31], and several other NMD genes have been shown to be significantly associated with neurodevelopmental disorders [18, 32].

To understand the underlying mechanism by which NMD influences and controls biological events, there has been a large effort to identify RNAs targeted for decay by NMD. Hundreds of both putative and high-confidence NMD-target RNAs have been defined by different studies [33–39]. The assumption has generally been that intrinsic features of a given mRNA determine whether or not it is degraded by NMD. This notion has been fueled by the discovery of specific signatures on mRNAs that elicit their decay by NMD. The most robust NMD-inducing feature is the presence of at least one exon–exon junction downstream of the stop codon defining the main open reading frame (ORF) [4–6, 40, 41]. This NMD-inducing feature is driven by the EJC, which interacts with NMD factors to promote RNA decay upon translation termination [3]. Other NMD-inducing features include a long 3′ untranslated region (UTR) and the presence of a short upstream ORF (uORF) upstream of the main ORF [4–6, 40–41]. While these two features have been shown to be capable of triggering NMD [4], these features are not robust. There is no specific length of 3′ UTR that triggers NMD [42] and most uORFs must not elicit NMD given that a much larger fraction of mammalian mRNAs have uORFs than those degraded by NMD [43].

In this report, we address the hypothesis that cellular context is another critical determinant as to whether or not an mRNA is degraded by NMD. This hypothesis has not been previously directly addressed, as, to our knowledge, no previous study has directly compared high-confidence NMD-target mRNAs—genome-wide—in different cell types. Moreover, caveats associated with many prior studies identifying NMD-target mRNAs have clouded interpretation of the results. First, a large number of NMD-target mRNAs have been identified in immortalized and malignant cell lines [33–34, 36–39, 44–46], raising doubts as to physiological relevance. Second, several studies have defined NMD-target mRNAs using only a single criterion—upregulation in response to NMD deficiency [9, 47]. Many (or even most) such mRNAs can be upregulated due to indirect regulation by NMD. Third, some of the approaches used to screen for direct NMD targets are subject to artifact, such as using toxic transcriptional inhibitors to measure RNA half-life [8, 48]. Thus, while a large number of putative NMD-target mRNAs have been identified by the field, the degree of confidence that these are *bona fide* NMD targets is variable. Here, we use multiple approaches to define high-confidence NMD targets in normal primary cells and tissues and find that, surprisingly, most direct NMD-target mRNAs are cell type- and tissue-specific. Our results imply that the presence of an NMD-inducing signal on an mRNA is not sufficient to trigger its decay; that cellular context is a critical determinant as to whether or not an mRNA with an NMD-inducing signal is degraded.

Another question we address in this report is the functional relationship of NMD factors. The current dogma in the field is that there are two types of NMD factors: “core factors” and “branch factors,” the latter of which promote the decay of only a subset of the mRNAs degraded by the core factors [15, 49]. A candidate NMD-branch factor is UPF3B, an adaptor protein that bridges UPF2 with the EJC, and also has a mid-domain that promotes NMD by an unknown mechanism [4–6]. Numerous studies have shown that either depletion or complete loss of UPF3B upregulates NMD-target mRNAs in cultured mammalian cells [9, 31, 44, 45, 50–53], mouse tissues *in vivo* [50, 54], and mouse cell types directly purified from *in vivo* [20, 54]. While UPF3B has never been definitively shown to be an NMD-branch factor, UPF3B has been thought to act in this manner because its knockdown (KD) in cultured cells [9] and *in vivo* [54] only upregulates some NMD-target mRNAs. Even complete loss of UPF3B—either in cultured cells [44, 45, 54] or *in vivo* [20, 21, 54]—only upregulates a relatively small number of NMD-target mRNAs. Further evidence that UPF3B is an NMD-branch factor is the finding that mice with a null mutation in *Upf3b* are viable and fertile [21], while mice with mutations in several other NMD factor genes, including *Upf1*, *Upf2*, *Upf3a*, *Smg1*, or *Smg6*, suffer from early embryonic lethality [22–24, 55]. Likewise, zebrafish embryos depleted of UPF1, UPF2, SMG5, SMG6, or SMG7 have severe developmental defects, whereas those depleted of UPF3B do not [56].

Instead of being essential for early embryonic development, UPF3B has key roles in the nervous system. In humans, nonsense and missense mutations in the lone X-linked *UPF3B* gene in males cause intellectual disability [18, 31, 57]. In addition, *UPF3B* mutations in humans are associated with schizophrenia, autism spectrum disorder, and attention deficit-hyperactivity disorder (ADHD) [18, 31]. In mice, null mutations in the *Upf3b* gene cause specific behavioral defects, including fear-conditioned learning deficits [21]. The underlying defect(s) responsible for these behavioral abnormalities in UPF3B-deficient mice and human is not known, but studies have shown loss or depletion of UPF3B impairs neural differentiation *in vitro* and dendritic spine maturation *in vivo* [21, 51, 58]. At the molecular level, it is hypothesized that UPF3B mediates its effects on the nervous system by promoting the decay of a small number of NMD-target mRNAs whose downregulation is critical for normal nervous system development and function [20, 21, 51–55]. In this communication, we report the identification of mRNAs targeted for decay by UPF3B in a physiologically relevant cell type—neural progenitor cells (NPCs). Intriguingly, we find that these UPF3B-dependent NMD-target transcripts in NPCs are largely different than those targeted for decay by UPF3B in embryonic stem cells (ESCs). We also make the surprising discovery that the downregulation of most of these mRNAs by UPF3B is not perturbed by depletion of the NMD core factor UPF2. This raises questions about the relationship of core and branch NMD factors. Coupled with our finding that NMD-target mRNAs are primarily defined in a cell type-specific manner, our results suggest that the current view of NMD as a hierarchical and rigid RNA turnover pathway requires revision.

## Materials and methods

### Mice

This study was carried out in strict accordance with the guidelines of the Institutional Animal Care and Use Committee (IACUC) at the University of California, San Diego. The protocol was approved by the IACUC at the University of California, San Diego (permit number S09160). All mice were housed under a 12 h light:12 h dark cycle and provided with food and water *ad libitum*.

### Cell lines

H9 human ESCs (WA09) were maintained in StemFlex^TM^ medium (Gibco) on plates coated with human ESC-qualified Matrigel (Corning). For expansion, colonies were split (1:10 ratio) after Accutase (Gibco) treatment. Mycoplasma testing was carried out routinely using the MycoAlert^®^ mycoplasma detection kit (Lonza).

As described previously [59], for mesoderm differentiation, human ESCs were harvested and seeded as single cells at 1 × 10^4^ cells per cm^2^. After 24 h, the media were replaced with STEMdiff™ mesoderm induction medium (STEMCELL Technologies) for 4 days. For endoderm differentiation, human ESCs were harvested and seeded as single cells at 1 × 10^4^ cells per cm^2^. After 24 h, the media were replaced with DMEM:F12/RPMI (1:1) medium (Gibco) supplemented with NaHCO_3_ (2.5 μg/ml; Sigma), 1% GlutaMAX (Gibco), glucose (5.5 mM; Sigma), 0.1% FAF–BSA (Sigma), and ITS:X (1:100; Gibco) supplemented with WNT3A (20 ng/ml), BMP4 (10 ng/ml; R&D Systems), and Activin A (100 ng/ml) for 1 day, followed by incubation for 3 days with the same media containing Activin A (100 ng/ml) only.

NPCs were generated from human ESCs as described previously [60]. Once 80% confluent, human ESCs were cultured in DMEM/F12 media (Gibco) supplemented with N2 (1:100; Gemini), 5 μM Rock inhibitor, 1 μM Dorsomorphin, and 10 μM SB431542 (STEMCELL Technologies) for 2 days. To generate embryoid bodies, the colonies were then cut into a big chess pattern, and cultured in DMEM/F12 media (Gibco) supplemented with N2 (1:100; Gibco), 1 μM Dorsomorphin, and 10 μM SB431542 for 5 days with rotation (95 rpm) inside a 37°C incubator. The embryoid bodies were then replated on plates coated with human ESC-qualified Matrigel (Corning) in DMEM/F12 media supplemented with N2 (1:200; Gemini) and Gem21 (1:100; Gemini) for 7 days. Neural rosettes were picked and dissociated with Accutase (Gibco) and plated on plates coated with poly-ornithine/Laminin in DMEM/F12 media supplemented with N2 (1:200; Gemini), Gem21 (1:100; Gemini), and 20 ng/mL bFGF (Sigma). Homogeneous populations of NPCs were achieved after three to four passages with Accutase in the same condition.

For gene knockdown (KD) via small interfering RNAs (siRNAs) in human ESCs and NPCs, Lipofectamine™ RNAiMAX transfection reagent (Invitrogen) was used following the manufacturer’s instructions. Briefly, human ESCs and NPCs were lifted with Accutase (Gibco) and seeded onto Matrigel-coated plates supplemented with 10 μM Rock inhibitor. Typically, for one well in a 12-well plate, we seeded 100 000 cells and used 3 μl RNAiMAX plus 1 μl of 10 μM siRNA stock to make the transfection reagent mix according to the manufacturer’s instructions. Cells were harvested for analysis at 48 h after transfection. Scramble siRNA: Silencer™ Select Negative Control #1. HNRNPL siRNA: CGGAUGUUCUUUACACUAUtt. UPF2 siRNA: GCUCGGAAUUUUUAUGAGAtt.

### Generation of NMD-deficient stem cells

We took two steps to generate UPF2-inducible knockout (iKO) human ESCs:

Step 1: We introduced loxP sites into intron 1 and 2 of both alleles of *UPF2* in human ESCs. This was achieved using guide RNAs (gRNAs) targeting intron 1 (TAGGCCTAACTAATAATGAT) and intron 2 (TAGGCCTAACTAATAATGAT), designed using the CRISPOR online tool [61]. Annealed oligonucleotides containing these gRNA target sequences were cloned into the PX459 expression vector [62] for transfection. The donor DNA co-transfected with the gRNA plasmids was comprised of *UPF2* sequences harboring two LoxP elements within the gRNA target sequences, ordered from IDT. The right and left homology arms of the donor DNA consisted of ∼800 nt of *UPF2* sequences upstream of the 5′ gRNA sequence and downstream of the 3′ gRNA sequence, respectively [polymerase chain reaction (PCR) amplified from human genomic DNA]. These three fragments were then assembled by PCR [63] and the sequence verified by Sanger sequencing before transfection.

Step 2: To introduce Cre-ERT into the *AAVS1* locus of ESCs, a gRNA targeting *AAVS1* intron 1 (TATAAGGTGGTCCCAGCTCG) was used. Annealed oligonucleotides containing this gRNA target sequence were cloned into the PX459 expression vector [62] for transfection. The donor DNA co-transfected with the gRNA plasmid contained Cre-ERT2 (derived from the pCAG-CreERT2 vector [64]) and right and left homology arms (∼1 kb in length) derived from the *AAVS1* locus (PCR amplified from genomic DNA).

To generate UPF3B-KO human ESC clones, two gRNA sequences targeting *UPF3B* intron 1 (ATAACTTAGTAAGCCAACGC) and intron 2 (AATTATGTAAATGTACTCGC) were designed using the CRISPOR online tool [61]. Annealed oligonucleotides containing these gRNA target sequences were cloned into the PX459 expression vector [62] for transfection.

The gRNA expression vectors and donor DNA described above were transfected into human H9 ESCs using the Nucleofector^TM^ kit (Lonza), following the manufacturer’s instructions. Single-cell colonies were picked and genotyped. Karyotyping was performed using an Illumina BeadChip platform.

### Quantitative real-time reverse transcription PCR (qRT-PCR) analysis

Total cellular RNA was isolated using TRIzol (Invitrogen), as previously described [65]. Reverse transcription-PCR analysis was performed using 1 μg of total cellular RNA using the iScript™ cDNA synthesis kit (Bio-Rad), followed by PCR amplification using SYBR Green (Bio-Rad) [66] and the ΔΔCt method (with ribosomal L19 for normalization). The primers are listed in Supplementary Table S7. Statistical significance was determined using the paired Student’s *t*‐test.

### Immunofluorescence analysis

NPCs were fixed in 4% paraformaldehyde for 20 min on ice, and blocked with 0.05% Triton X-100 (Sigma) and 5% donkey serum (Sigma) in phosphate-buffered saline (PBS) at room temperature for 1 h. Cells were then incubated overnight with the primary antibody PAX6 (1:100; Proteintech #12323-1-AP) at 4°C and incubated with secondary antisera (Alexa Fluor^®^ 488) for 1 h at room temperature. The nuclei were counterstained with DAPI (Vector Laboratories). The images were viewed using a Leica DMI4000 B fluorescence microscope, as described previously [67, 68].

### Annexin V/PI staining and flow cytometry

Apoptosis and necrosis were assessed as described [69], using the Annexin V-FITC/PI apoptosis detection kit (Sigma) coupled with flow cytometry, following the manufacturer’s instructions. Briefly, cells were washed twice with PBS and then co-stained with Annexin V-fluorescein isothiocyanate (FITC) and the nonvital dye propidium iodide (PI), which allowed the discrimination of viable cells (FITC − PI −), early apoptotic cells (FITC + PI −), and necrotic cells (FITC + PI +) when analyzed by fluorescence-activated cell sorting analysis (the gating was set based on unstained cells).

### Western blot analysis

Samples were incubated in radioimmunoprecipitation assay buffer (Bio-Rad) supplemented with protease inhibitor cocktail (Sigma) on ice for 30 min, followed by centrifugation at 16 000 × *g* for 15 min at 4°C. The lysates were then transferred to new tubes, and protein level was quantified using the DC™ Protein Assay kit (Bio‐Rad). Twenty micrograms of the protein samples were separated on polyacrylamide gels, and western blot analysis was performed as previously described [70]. The primary antibodies used were a rabbit anti-human UPF2 polyclonal antisera (a kind gift from Dr Jens Lykke-Andersen) [71], rabbit anti-human UPF3B antisera (Catalog# 23301-1-AP; Proteintech), rabbit anti-human PARP antisera (Catalog# 9532S; Cell Signaling Technology), and mouse anti-human GAPDH monoclonal antibody (Catalog# GT239; GeneTex). Quantification of the blots was performed using NIH ImageJ (1.8.0).

### RNA-seq processing and analysis

RNA sequencing (RNA-seq) was performed as described previously [2, 69, 70, 72]. Total RNA was extracted using the RNeasy Plus Mini kit (Qiagen), following the manufacturer’s protocol. Ribosomal RNAs were removed using the NEBNext^®^ rRNA Depletion Kit v2 (New England Biolabs). The enriched mRNA was then used to make the library using the SMART-Seq^®^ v4 PLUS Kit (Takara Bio), as per manufacturer’s instructions. Libraries were sequenced (pair-end reads) with an Illumina NovaSeq 6000 platform for 100 cycles at the UCSD institute for Genomic Medicine (IGM) core.

Reads were filtered for quality and aligned with STAR (2.5.2b) against the GRCh38 (Ensembl version 106) or GRCm39 (Ensembl version 105). The exon counts were aggregated for each gene to build a read count table using SubRead function featureCounts. Differentially expressed genes (DEGs) were defined using the DESeq2 program with the following thresholds: |Log_2_FC| >1 and *q* <0.01. To measure transcript (or isoforms) expression levels, we used the RSEM package [73]. Differentially expressed transcripts (DETs) were defined using the EBSeq program [74] with the following thresholds: PPEE <0.01 and PostFC >2 or <0.5.

To systematically analyze the isoform changes, we used IsoformSwitchAnalyzeR [75] with minor modifications to the standard workflow. We removed isoforms expressed at <0.1 transcripts per kilobase million (TPM) and those not contributing at least 5% of the total parent gene expression. After this filtering, only genes with at least two transcripts were kept. The statistical analysis of differential used transcripts was carried out using DEXSeq [76] with the *isoformSwitchTestDEXSeq* function. ORFs, NMD-sensitive isoforms, and alternatively-processed isoforms were identified and analyzed by IsoformSwitchAnalyzeR, as described [75]. We used the *analyzeSwitchConsequences* function to analyze isoform switches for the following: intron retention, NMD sensitivity, alternative transcription start and termination sites, changes in the last exon, changes in isoform length, and exon number.

The R package program, pheatmap, was used for clustering and generating heatmap plots. The Metascape platform [77] and R package program, clusterProfiler [78], were used for gene functional annotation using the following parameters: *p *<0.01, minimum count of 3, and enrichment factor >1.5.

The analysis of NMD-inducing features was performed as previously described [20], using a previously published in-house Python script [79]. Only Ensembl transcripts with a detectable 5′ UTR and 3′ UTR were considered for subsequent analysis. Transcripts were considered to have a “downstream exon junction” (dEJ) if they had at least one exon–exon junction ≥50 nt downstream of stop codon defining the main ORF. Transcripts were considered to have an “uORF” if the ORF upstream of the main ORF was ≥30 nt long and contained an initiator codon in a context with a purine at the -3 position and/or a guanine at the +1 position [relative to the A in the AUG initiation codon (+1)], both of which are key Kozak consensus sequence critical for initiating translation [80]. To reduce the probability of identifying uORFs that can re-initiate translation (and thus escape NMD), one additional criterion we used is the uORF must not contain any sequences in the main ORF. 3′ UTR length was calculated using the APAtrap program [81].

To identify genes with highly correlated expression patterns across different cell types, we used module preservation analysis within the Weighted Gene Co-Expression Network Analysis (WGCNA) framework [82]. This framework constructs a gene co-expression network using Pearson correlation, grouping genes with similar expression patterns into distinct modules or clusters. To assess whether the density and connectivity patterns of these modules from a reference dataset are preserved in a test dataset, we calculated the Zsummary statistic. A Zsummary value >2 was used as the cutoff to indicate significant module preservation.

### RNA immunoprecipitation sequencing analysis

Phosphorylated UPF1 (pUPF1) RNA immunoprecipitation sequencing (RIPseq) was performed as described previously [83], with minor modifications. Briefly, 15 million human ESCs or NPCs were incubated in 50 nM okadaic acid for 3 h. Given that extended okadaic acid treatment may inhibit translation [84, 85]; we used a lower concentration (50 nM) compared with the 200 nM used previously [83] to minimize this potential issue. Cellular RNAs bound by pUPF1 were immunoprecipitated using anti-pUPF1 [anti-phospho-UPF1(Ser1127)] and Dynabeads protein A magnetic beads (Thermo Fisher Scientific). The bead-bound RNA–pUPF1 complexes were incubated for 20 min at 4°C with 1 U/μl RNase I (Ambion) to digest nonbound RNA. The bead-bound RNA–pUPF1 complexes were extensively washed and eluted using immunoprecipitation elution buffer [125 mM Tris–HCl (pH 6.8), 4% sodium dodecyl sulfate, 20% glycerol, 0.1% bromophenol blue, 10% 2-mercaptoethanol]. The RNA was purified using TRIzol reagent followed by ethanol precipitation. In parallel, control immunoprecipitations (IPs) using rabbit IgG were performed. Additional control samples (input) were prepared that did not undergo the IP process. The purified RNA fragments were used to make the library using the NEBNext Ultra II Directional Library Prep Kit (New England Biolabs), as per manufacturer’s instructions. Libraries were sequenced (pair-end reads) with an Illumina NovaSeq 6000 platform for 100 cycles at the UCSD IGM core.

Reads were filtered for quality and aligned with STAR (2.5.2b) against GRCh38 (Ensembl version 106). Low-quality reads (minimum quality threshold of 30) were discarded (at least 50% of bases were above this quality threshold). PCR duplicates was removed using BEDTools [86] and the reads were re-aligned and peak calling was performed using the CLAM program [87] to compare the signal of pUPF1 RIP samples with the input samples. Peaks with >1-fold enrichment (*q* <0.01) were considered positive. Peak annotation to mRNAs (5′ UTR, coding DNA sequence [CDS], and 3′ UTR) was performed using the BEDTools *intersect* function [86]. Peaks located in 3′ UTRs were extracted and used to identify motifs with the Homer *findMotifsGenome.pl* function [88], with the motif length set to ≤8 nt. Track screen shots were produced in IGV (version 2.6.2) [89].

### Statistical analysis

Graphs were generated using GraphPad Prism 10 Project or Microsoft Office Excel. All numerical data are presented as mean ± standard error of the mean (SEM) or mean ± standard deviation, as needed. Differences between groups were compared by *t*-test or two-way ANOVA.

## Results

### Generation of inducible NMD-deficient human ESCs

**Figure 1.**
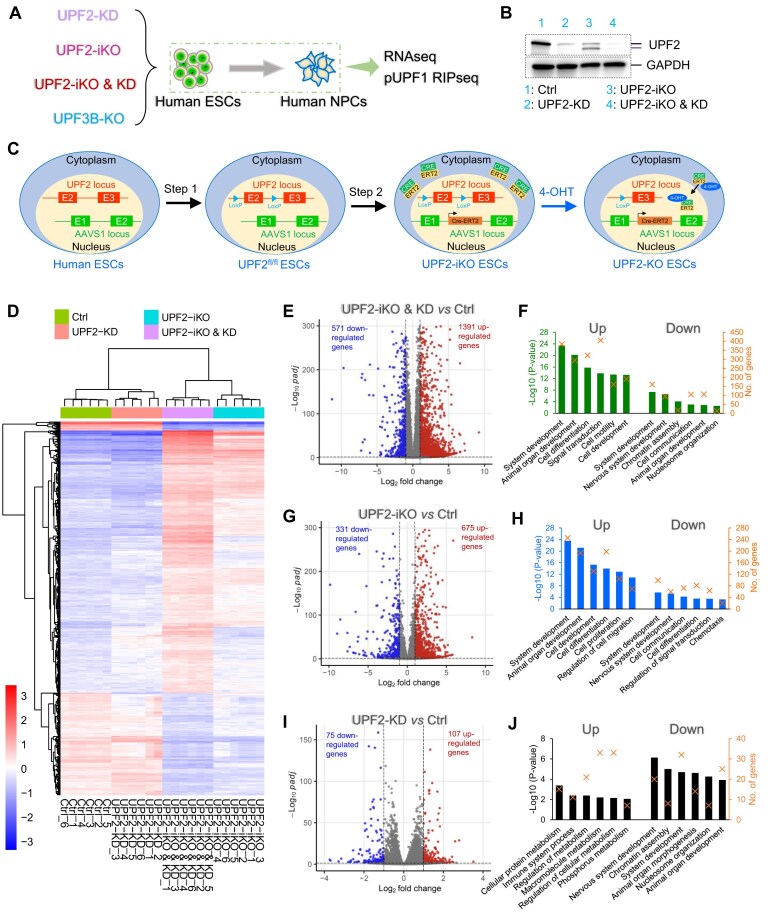
Identification of UPF2-regulated genes in human ESCs. (**A**) The strategy we used to define NMD-target mRNAs in primary stem cells. UPF2-dependent NMD-target mRNAs in ESCs were identified by UPF2 KD with a UPF2 siRNA; see Supplementary Fig. S1) and by using an inducible system to transiently KO UPF2 (*UPF2*-iKO; described in panel C). Transcripts upregulated in response to these UPF2 disruptions were defined as “high-confidence” NMD-target mRNAs if they harbored a robust NMD-inducing feature. If such transcripts also exhibited high occupancy of pUPF1 (determined by RIPseq analysis), we defined them as “unambiguous” NMD-target mRNAs. UPF3B-dependent NMD-target mRNAs were identified by knocking out UPF3B (*UPF3B*-KO) in ESCs and then performing the same analysis as described above. UPF2- and UPF3B-dependent NMD-target mRNAs in NPCs were identified by the same approaches as described above in control (Ctrl) and mutant NPCs derived from the ESCs described above. (**B**) Western blot analysis of ESCs cultured under the indicated conditions. *UPF2*-iKO generates a truncated version of UPF2. GAPDH is the loading Ctrl. (**C**) *UPF2*-iKO scheme. Step 1: LoxP elements were introduced on either side of *UPF2* exon 2 in ESCs. Step 2: A Cre-ERT2 construct was introduced into the *AAVS1* safe harbor site. 4-OHT treatment triggers Cre-ERT2 nuclear translocation and consequent exon 2 deletion. (**D**) Unsupervised hierarchical clustering of RNA-seq samples from four groups of ESCs: (i) UPF2^fl/fl^ ESCs transiently transfected with siUPF2 (“UPF2-KD”), (ii) UPF2^fl/fl^ ESCs transiently transfected with a scramble siRNA (“Ctrl”), (iii) UPF2^fl/fl^ Cre-ERT2 ESCs transiently transfected with UPF2 siRNA (“UPF2-iKO & KD”), and (iv) UPF2^fl/fl^ Cre-ERT2 ESCs transiently transfected with scramble siRNA (“UPF2-iKO”). Two independently derived UPF2^fl/fl^ or UPF2^fl/fl^ Cre-ERT2 ESC clones were analyzed, with three experimental replicates for each (e.g. samples 1–3 were from clone 1, and samples 4–6 were from clone 2). All ESCs were incubated with 4-OHT. (**E**, **G**, and **I**) DEGs (*q* <0.01, fold change >2) from each comparison. (**F**, **H**, and **J**) Biological functions associated with the DEGs defined in the panel to the left. Statistical significance [−Log_10_ (*p*-value)] is indicated by the bar. The number of DEGs for a given category is indicated by an “X.”

We elected to define NMD-target mRNAs using an inducible system to enrich for direct targets. By contrast, long-term NMD deficiency likely favors upregulated indirect targets (e.g. mRNAs upregulated in response to the action of direct NMD targets) and it can lead to physiological changes in cells, including induction of the unfolded protein response, toxicity, and even cell death [90, 91]. To focus on physiologically relevant NMD-target mRNAs, we chose to use normal primary cells rather than immortalized and malignant cell lines. We chose to perturb NMD by depleting the core NMD factor, UPF2, since this approach has been widely used to elicit NMD deficiency both *in vitro* and *in vivo* [24, 25, 47, 92–96]. Furthermore, UPF2 appears to be limited in its action to NMD, whereas the primary NMD core factor, UPF1, acts in several different RNA turnover pathways in addition to NMD [4–6, 40]. Finally, to more fully capture NMD-target mRNAs, we perturbed UPF2 by different approaches (Fig. 1A). The logic behind this is as follows: Approaches that strongly perturb NMD will have the advantage of potentially capturing even weak NMD-target mRNAs, but will have the disadvantage of likely increasing the proportion of upregulated indirect target mRNAs, including those involved in stress responses [90, 91]. Approaches that only modestly perturb NMD will likely avoid this disadvantage and, furthermore, such approaches will be less likely to induce the feedback regulatory response that increases the levels of several NMD factors to compensate for perturbations in NMD [54, 97]. Finally, increasing evidence suggests that NMD is not a simple monolithic pathway [49], which, if true, means that defining its scope of action requires multiple approaches.

To perturb UPF2, we chose to use two approaches, implemented either singly or together: (i) transient KD and (ii) iKO. For the former, we chose to transiently transfect an siRNA targeting UPF2. Screening of three UPF2 siRNAs revealed that siRNA #3 was the most effective in reducing *UPF2* mRNA level (by ∼80%; Supplementary Fig. S1A). This siRNA also reduced the level of UPF2 protein (Fig. 1B), and thus, we chose it for our follow-up experiments below.

To inducibly KO *UPF2*, we created human ESCs that acquire a debilitating deletion in the *UPF2* gene in response to an exogenous signal. We generated these UPF2-iKO ESCs in two steps (Fig. 1C). First, we introduced loxP sites on either side of *UPF2* exon 2 using a dual single-guide RNA (sgRNA) targeting strategy. We choose to target exon 2, as its deletion has been shown to inactivate NMD in a wide variety of cell types *in vivo* [24, 25, 47, 92–96]. Two independent clones of these UPF2^fl/fl^ ESCs were generated. Second, to induce exon 2 deletion in these cells, we stably inserted into these two cell clones a Cre-ERT2 fusion construct encoding a form of Cre recombinase that translocates into the nucleus to generate loxP-dependent deletions in response to 4-hydroxytamoxifen (4-OHT). Using homologous recombination, this Cre-ERT2 fusion construct was inserted into the *AAVS1* locus, a well-known human “safe harbor” that has the ability to maintain the expression of an exogenously inserted gene [98]. Both the UPF2^fl/fl^ and UPF2^fl/fl^ Cre-ERT2 cells exhibited normal pluripotency, based on marker gene expression (Supplementary Fig. S1B).

Exon 2 harbors the *UPF2* start ATG and thus exon 2 deletion should lead to the inability to translate full-length UPF2. Indeed, western blotting analysis showed that full-length UPF2 protein was reduced in level after 4-OHT treatment (Fig. 1B). 4-OHT treatment also led to production of a truncated protein that may arise due to an initiator codon downstream of the normal initiator codon in exon 2 (Fig. 1B). A truncated UPF2 protein was also observed when *Upf2* exon 2 was deleted in mice *in vivo* by a previous study [47]. Treatment with 4-OHT for 24 h was sufficient to modestly reduce UPF2 levels (Supplementary Fig. S1C) and thus was worth consideration for downstream analysis because this early time point might minimize indirect effects (e.g. upregulation of non-NMD-target mRNAs). However, we chose to, instead, perform downstream analysis on cells treated with 4-OHT for 48 h because this longer treatment more strongly reduced the level of full-length UPF2 protein (Fig. 1B) and thus would probably capture more NMD-target mRNAs. Of note, 48 h 4-OHT treatment did not significantly induce cell apoptosis or necrosis (Supplementary Fig. S1D and E).

### Identification of UPF2-regulated genes in human ESCs

As a first step to define NMD-target mRNAs in human ESCs, we identified UPF2-regulated genes in these cells using bulk RNA-seq analysis. Two independent UPF2^fl/fl^ and UPF2^fl/fl^ Cre-ERT2 ESC clones were tested (four ESC clones total; three experimental replicates for each). Four conditions were tested: Ctrl, UPF2-iKO, UPF2-KD, and UPF2-iKO & KD. Hierarchical clustering showed that replicates of each group were closely aligned, while samples from the different groups were well separated (Fig. 1D).

DEG analysis showed that the condition that elicited the strongest reduction in full-length UPF2 protein levels—UPF2-iKO & KD (Fig. 1B)—also dysregulated the largest number of genes (Fig. 1E). A total of 1962 genes were significantly dysregulated, with the majority (71% [1391/1962]) upregulated (*q* <0.01, |log_2_FC| >1; Fig. 1E and Supplementary Table S1), consistent with UPF2 depletion leading to NMD deficiency. The 1391 upregulated genes are enriched for “system development,” “cell differentiation,” “signal transduction,” and “cell motility” functions, while the 571 downregulated genes are enriched for “nervous system development,” “chromatin assembly,” “cell communication,” and “nucleosome organization” functions (Fig. 1F).

The UPF2-iKO condition dysregulated 1006 genes (*q* <0.01, |log_2_FC| >1; Fig. 1G and Supplementary Table S1). Like the UPF2-iKO & KD condition, the UPF2-iKO condition upregulated more genes (675) than it downregulated (331). The ∼2:1 upregulated-to-downregulated DEG ratio suggests that the majority of these genes correspond to direct NMD-target mRNAs. The genes up- and downregulated by the UPF2-iKO conditions are enriched for similar functions (Fig. 1H) as the UPF2-iKO & KD condition (Fig. 1F).

The UPF2 KD condition alone reduced both *UPF2* mRNA and UPF2 protein level by ∼80% (Fig. 1B and Supplementary Fig. S1A), but only dysregulated 182 genes (*q* <0.01, |log_2_FC| >1; Fig. 1I and Supplementary Table S1). While a relatively small number of genes were dysregulated, a high proportion of these genes are likely to be directly regulated by NMD (as opposed to being regulated as a result of toxicity caused by NMD loss), given that the UPF2 siRNA would not be expected to completely eliminate UPF2. Furthermore, the UPF2 siRNA did not elicit detectable apoptosis (Supplementary Fig. S1D and E). As with the other two conditions, the UPF2-KD condition upregulated more genes (107) than it downregulated (75). The upregulated genes are enriched for some unique functions not enriched by the other two conditions (e.g. “metabolic process” and “immune system process”) (Fig. 1J). Interestingly, a common category enriched in downregulated genes by all three conditions is “nervous system development” (Fig. 1F, H, and J; see Supplementary Table S1 for a list of the “nervous system development” genes).

### Identification of NMD-target mRNAs in human ESCs

To identify NMD-target mRNAs in human ESCs, we performed transcript analysis (rather than the gene analysis described above). To achieve this, we used two pipelines: (i) the RSEM program that quantifies transcript steady-state levels from RNA-seq data [73], and (ii) the IsoformSwitchAnalyzeR program that specifically identifies regulated alternatively processed mRNAs [75], a common target of the NMD pathway [26, 99, 100]. We then stratified the upregulated transcripts defined by these programs into different categories based on known NMD-inducing features.

#### Approach 1: identification of NMD-target transcripts harboring NMD-inducing features in ESCs

**Figure 2.**
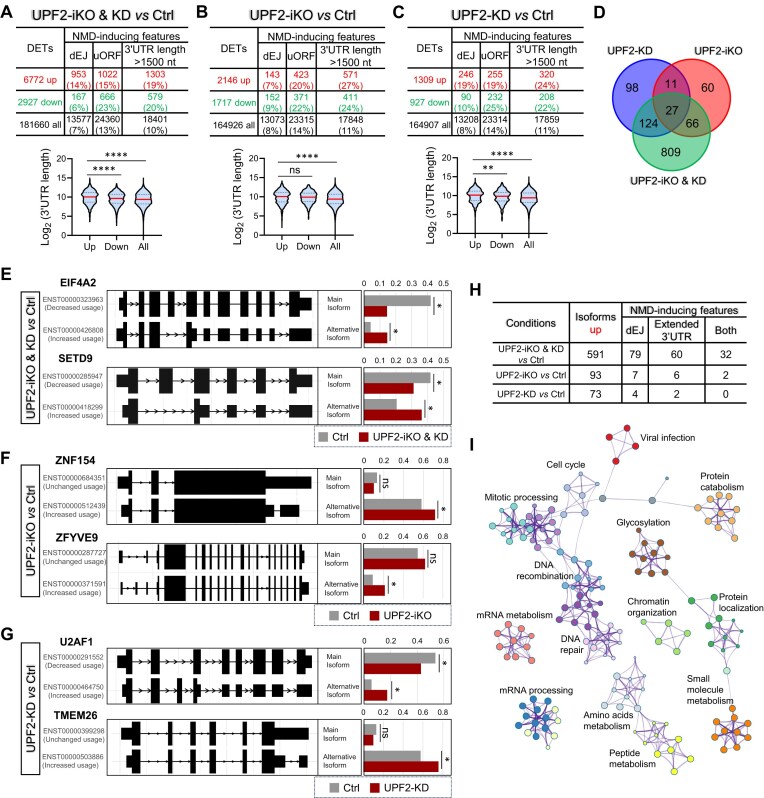
Identification of high-confidence NMD-target transcripts in human ESCs. (**A**–**C**) Top panel: The total number of DETs, as well as the number of DETs with the indicated NMD-inducing feature, for the UPF2 disruption condition shown (determined from the datasets described in Fig. 1). Bottom panel: Violin plot showing the 3′ UTR length of the up- and down-regulated DETs, as well as all transcripts. ** *p* <0.01; **** *p* <0.0001; ns, not statistically significant (unpaired t-test). (**D**) Overlap amongst high-confidence UPF2-dependent NMD-target RNAs (those with a dEJ and/or an extended 3′ UTR) identified from the datasets in panels (A)–(C). (**E**–**G**) Examples of alternatively processed transcripts degraded by NMD in ESCs, as defined by the IsoformSwitchAnalyzeR program. The bar graphs show the expression of the main isoform versus an alternative isoform harboring an NMD-inducing feature that is upregulated by the indicated UPF2 disruption (other isoforms are not shown). The value shown for each isoform is the proportion of the total. Bonferroni-adjusted *t*-test * *q*<0.05. (**H**) Alternatively processed mRNA isoforms upregulated by the indicated UPF2 perturbation condition, as defined by the IsoformSwitchAnalyzeR program. Shown is the total number of upregulated transcripts, as well as upregulated transcripts with the indicted NMD-inducing feature. “Both” refers to upregulated transcripts with both a dEJ and an extended 3′ UTR. (**I**) Biological functions defined by the Metascape program (*p* <0.01) associated with the full profile of high-confidence UPF2-dependent NMD-target mRNAs in ESCs [defined from the datasets shown in panels (A)–(C) and (H)].

Use the RSEM program [73], we found that the UPF2-iKO & KD, UPF2-iKO, and UPF2-KD conditions significantly altered the expression of 6722, 2146, and 1309 transcripts, respectively (FDR <0.01, |log_2_FC| >1; Fig. 2A–C). We then determined what proportion of the transcripts upregulated by these conditions have known NMD-inducing features. The NMD-inducing feature that most consistently triggers NMD is a “dEJ,” an exon–exon junction >50-nt downstream of the stop codon defining the main ORF [41]. Given that a dEJ is a robust NMD-inducing feature that is widely used to predict NMD targeted mRNAs [20, 24, 33–35, 39, 101], upregulated transcripts harboring a dEJ are very strong candidates to be direct NMD-target mRNAs. The UPF2-iKO & KD, UPF2-iKO, and UPF2-KD conditions upregulated 953, 143, and 246 such dEJ+ transcripts, respectively (Fig. 2A–C). As support that these are direct NMD targets, the dEJ feature is enriched in transcripts upregulated in response to UPF2-iKO & KD or UPF2-KD alone (Fig. 2A and C). For example, 14% of UPF2-iKO & KD-upregulated transcripts harbored a dEJ, whereas only 6% of downregulated transcripts in UPF2-iKO & KD cells harbored a dEJ (Fig. 2A). In contrast, UPF2-iKO cells did not have a greater proportion of dEJ+ upregulated transcripts than dEJ+ downregulated transcripts (Fig. 2B), raising the possibility that this condition instead enriches for other classes of NMD-target mRNAs. Together, these analyses identified dEJ-containing mRNAs that are negatively regulated by UPF2 and thus high-confidence NMD-target mRNAs in human ESCs.

We next considered another NMD-inducing feature—a long 3′ UTR. A number of studies have demonstrated long 3′ UTRs can trigger NMD [36, 42, 93, 102, 103], but given that no particular 3′ UTR length induces NMD, this feature would not necessarily be predicted to be enriched in upregulated transcripts. Indeed, the frequency of upregulated transcripts containing long 3′ UTRs (>1500 nt) is only modestly increased in frequency for the UPF2-iKO and UPF2-KD conditions; and there was no change in this frequency for the UPF2-iKO & KD condition (Fig. 2A–C). However, we found that the average length of 3′ UTRs is significantly longer in upregulated transcript versus downregulated transcripts for all three conditions (Fig. 2A–C), providing correlative support for the importance of this NMD-inducing feature in human ESCs.

To define transcripts that are candidates to be degraded by long 3′ UTR-dependent NMD, we modified our NMD-inducing feature criteria so as to only identify upregulated transcript isoforms with a 3′ UTR longer than the main transcript isoform from the same gene (>1000 nt, fold change >1.5). Several transcripts obey these criteria: 191, 45, and 49 transcripts from the UPF2-iKO & KD, UPF2-iKO, and UPF2-KD conditions, respectively (Supplementary Table S1). Interestingly, the majority of these alternatively processed transcripts with an extended 3′ UTR also have a dEJ (118/191, 24/45, and 35/49 transcripts upregulated in the UPF2-iKO & KD, UPF2-iKO, and UPF2-KD conditions, respectively). It remains to be determined whether these are degraded by NMD through one or both of these features. Regardless, analysis of the presence of “an extended 3′ UTR” is a potentially useful approach to identify NMD-target mRNAs.

We also examined uORF frequency in the upregulated transcripts, as uORFs in a small number of transcripts have been shown to trigger NMD [104, 105]. However, uORFs are widely present in mammalian mRNAs [43] and thus most uORFs probably do not elicit NMD. This leads to the prediction that transcripts upregulated by UPF2 depletion will not be enriched for uORFs. Indeed, the percentage of uORF+ upregulated transcripts is not higher than the percentage of uORF+ downregulated transcripts for any of the three UPF2-depletion conditions (Fig. 2A–C). Given this information, coupled with the fact that uORFs are not known to be a predictable NMD-inducing feature, we did not attempt to define uORF-dependent NMD targets.

In total, RSEM analysis identified a total of 1195 high-confidence NMD-target transcripts (from 932 genes) that are upregulated in response to UPF2 perturbation and have (i) a dEJ, (ii) an extended 3′ UTR, or (iii) both (Fig. 2D).

#### Approach 2: identification of alternatively processed NMD-target RNAs in human ESCs

To identify more transcripts targeted by NMD, we used IsoformSwitchAnalyzeR, a pipeline that specifically detects reciprocal changes in the level of alternatively processed mRNA isoforms [75]. This pipeline identified 655, 120, and 87 genes expressing at least 1 mRNA isoform undergoing a statistically significant change in level in response to the UPF2-iKO & KD, UPF2-iKO, and UPF2-KD condition, respectively (Fig. 2E–G; Supplementary Table S1). Given that only upregulated mRNAs are candidate NMD-target mRNAs, we focused on these; fitting this criterion were 591, 93, and 73 mRNA isoforms (from 564, 90, and 73 genes, respectively) in response to UPF2-iKO & KD, UPF2-iKO, and UPF2-KD conditions, respectively. Fig. 2H shows the proportion of these transcripts that have a dEJ and/or a 3′ UTR longer than in the main isoform. The percentage of upregulated transcripts with a dEJ and/or extended 3′ UTR is modestly higher for the UPF2-iKO & KD condition (18%; 107/591) than for the UPF2-iKO (12%; 11/93; *p*= 0.136) or the UPF2-KD (8%; 6/73; *p*= 0.034) conditions. Fig. 2E–G provide examples of the types of alternatively processing undergone by the upregulated isoform versus the main isoform, along with their degree of regulation.

The total number of upregulated alternatively processed transcripts with a dEJ and/or extended 3′ UTR identified by our approach 2 analysis, above, is 118 (after removing those upregulated by >1 condition). Forty-one of these 118 transcripts overlap with those identified using approach 1. Thus, the combination of both approaches allowed us to identify 1272 (1195 + 118 − 41) high-confidence NMD-target RNAs. These 1272 transcripts are statistically enriched for several biological functions, including “cell cycle,” “chromatin organization,” “DNA recombination and repair,” “RNA processing,” “RNA metabolism,” “peptide metabolism,” and “protein localization” (Fig. 2I).

### Identification of UPF3B-dependent NMD-target RNAs in human ESCs

**Figure 3.**
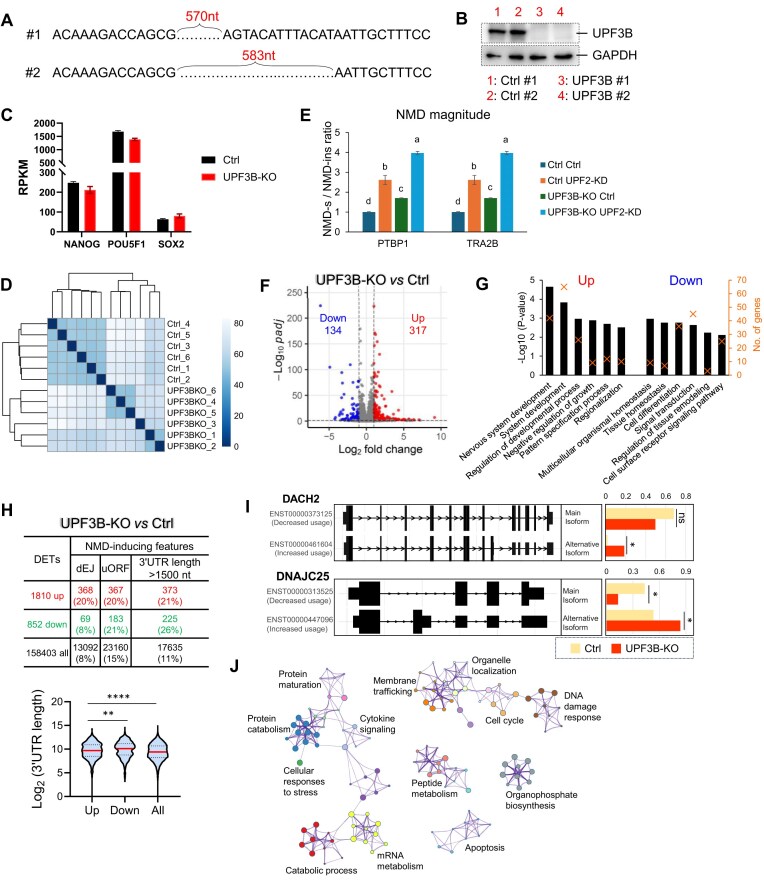
Identification of high-confidence UPF3B-dependent NMD-target transcripts in human ESCs. (**A**) The location of the deletion boundary on either side of *UPF3B* exon 2 in the two independent *UPF3B*-KO human ESC clones generated using CRISPR. (**B**) Western blot analysis of two independent clones of *UPF3B*-KO and Ctrl ESCs. Shown is a representative result from two independent experiments. (**C**) Expression of pluripotency genes, based on RPKM (reads per kilobase per million mapped reads) values from RNA-seq analysis. There is no statistical significant difference (Wald test) in the expression of the three pluripotency genes in *UPF3B*-KO versus Ctrl ESCs. (**D**) Unsupervised hierarchical clustering of the indicated ESCs assayed by RNA-seq (two independent Ctrl and *UPF3B*-KO human clones, with three biological replicates from each). (**E**) Effect of combined UPF3B loss and UPF2 KD on NMD magnitude in ESCs, as quantified by NMD-sensitive/NMD-insensitive mRNA isoform ratio. Expression of these mRNA isoforms from the *PTBP1* and *TRA2B* genes [107] was determined by quantitative PCR (qPCR) analysis of ESCs with the indicated genotype (Ctrl or *UPF3B*-KO) transiently transfected with a *UPF2* siRNA or a negative control scramble siRNA (Ctrl). The values shown are from two independently derived *UPF3B*-KO ESC clones, with two independent biological replicates. Statistical significance was determined using the Student’s *t*-test. Different letters (a, b, c, and d) denote statistically significant differences between different groups (*p* <0.05). (**F**) DEGs (*q* <0.01, fold change >2) identified from the RNA-seq analysis in panel (D). (**G**) Functions associated with the DEGs defined in panel (F). Statistical significance [−Log_10_ (*P*-value)] is indicated by the bar. The number of DEGs for a given category is indicated by an “X.” (**H**) Top panel: The number of DETs, as well as the number of DETs with the indicated NMD-inducing feature. Bottom panel: Violin plot showing the 3′ UTR length of the up- and down-regulated DETs, as well as all transcripts. ** *p* <0.01; **** *p* <0.0001 (unpaired t-test). (**I**) Examples of alternatively processed transcripts degraded by UPF3B-dependent NMD in ESCs, as defined by the IsoformSwitchAnalyzeR program. The bar graph shows the expression of the main isoform versus an alternative isoform harboring an NMD-inducing feature that is upregulated by the UPF3B loss (other isoforms are not shown). The value shown for each isoform is the proportion of the total. Bonferroni-adjusted *t*-test * *q*<0.05; ns, not statistically significant. (**J**) Biological functions defined by the Metascape program (*p* <0.01) associated with the full profile of high-confidence UPF3B-dependent NMD-target transcripts in ESCs.

There is considerable interest in UPF3B, particularly since mutations in the *UPF3B* gene cause intellectual disability and are associated with neurodevelopmental disorders in humans [18, 31, 57]. UPF3B is hypothesized to be an NMD-branch factor that is necessary for the decay of only a subset of the transcripts degraded by the entire NMD pathway [9, 20, 21, 44, 45, 49, 54]. To identify UPF3B-dependent transcripts in ESCs and to determine whether indeed UPF3B regulates a subset of NMD-target transcripts, we used the same loss-of-function/RNA-seq approach described above for UPF2. Since UPF3B is not required for cell line viability [44, 45] or even mouse or human viability [21, 31, 57], we knocked out UPF3B constitutively in human ESCs using the CRISPR–Cas9 system. Two independent *UPF3B*-KO human ESC clones were obtained (Fig. 3A and B). These clones had no obvious morphological differences from the ESCs they were derived from; they also exhibited normal pluripotency, based on marker gene expression as compared with Ctrl cells (Fig. 3C).

RNA-seq analysis of these two *UPF3B*-KO human ESC clones (three replicates per clone) and Ctrl human ESCs (six replicates) was performed (Fig. 3D). In agreement with previous studies showing that UPF3B regulates a small subset of NMD targets [9, 20, 21, 44, 45, 52, 54], only two of seven well-established NMD-target RNAs [8, 47, 54, 104, 106] were upregulated in response to *UPF3B* KO (Supplementary Fig. S1F). By comparison, five of these seven NMD targets were upregulated in response to *UPF2* KO and/or KD (Supplementary Fig. S1G). To assess whether UPF3B also acts as a “NMD amplifier,” as suggested by previous studies [9, 44–45, 55], we asked whether *UPF2* KD further upregulates the expression of NMD targets in *UPF3B*-KO ESCs. To rule out potential effects of transcription, we examined NMD-target mRNAs that have a corresponding non-NMD target isoform expressed from the same promoter, as defined by Zhao *et al.* [107]. As shown in Fig. 3E, UPF3B KO in combination with UPF2 KD more strongly upregulated these NMD targets than either condition alone, consistent with UPF3B acting as an NMD amplifier.

RNA-seq analysis revealed that more than twice as many genes are upregulated (317) than downregulated (132) in response to loss of UPF3B (Fig. 3F and Supplementary Table S2), consistent with UPF3B’s role as an NMD factor. These up- and downregulated genes are statistically enriched for several functions, including those shown in Fig. 3G. Given UPF3B’s known roles in neuronal development and normal brain function [18], it is notably that the most statistically enriched function associated with the upregulated genes is “nervous system development” (see Supplementary Table S2 for a list of these genes).

Using the RSEM-based approach described above for UPF2-regulated RNAs, we identified 1810 transcripts upregulated in response to loss of UPF3B (FDR <0.01, |log_2_FC| >1) (Supplementary Table S2). dEJs are enriched in these upregulated transcripts (20% dEJ+ versus 8% dEJ+ downregulated transcripts; Fig. 3H), consistent with a dEJ being a robust NMD-inducing feature [41]. In contrast, uORFs are not enriched in the upregulated transcripts (Fig. 3H). Likewise, long 3′ UTRs (>1500 nt) were not enriched (Fig. 3H) and the average length of the 3′ UTR is modestly shorter (rather than being longer if this feature consistently triggered NMD) in upregulated transcript versus downregulated transcripts. Together, this suggests that UPF3B may only rarely trigger NMD through long 3′ UTRs in human ESCs. To identify the few UPF3B-dependent NMD-target transcripts that are candidates to be regulated by long 3′ UTRs, we used the same approach as described above, for UPF2. This identified 88 upregulated transcripts with extended 3′ UTRs relative to the main isoform (Supplementary Table S2). A large proportion of these transcripts also had a dEJ (45/61).

In total, the above RSEM-based analysis identified 380 high-confidence UPF3B-dependent NMD-target RNAs (from 336 genes) that are upregulated in response to UPF3B loss and have a dEJ and/or an extended 3′ UTR.

Using the IsoformSwitchAnalyzeR pipeline [75] described above, we identified 90 genes encoding 138 RNA isoforms undergoing a significant alteration in isoform usage in *UPF3B*-KO versus Ctrl human ESCs (*q*<0.01) (Supplementary Table S2). Focusing our analysis on the 74 upregulated isoforms (derived from 74 distinct genes), 14 have a dEJ and 7 have an extended 3′ UTR relative to the main isoform (6 transcripts had both features; Fig. 3I and Supplementary Table S2). In total, 15 alternatively processed transcripts with a dEJ and/or extended 3′ UTR were defined using the IsoformSwitchAnalyzeR pipeline.

The total number of high-confidence UPF3B-dependent NMD-target transcripts (with a dEJ and/or an extended 3′ UTR) identified by the above approaches is 391. This is considerably less than the 1272 high-confidence NMD targets dependent on the core NMD factor UPF2 (Fig. 2), which is consistent with the hypothesis that UPF3B is an NMD-branch factor [9, 20, 21, 44–45, 49, 54]. These high-confidence UPF3B-dependent NMD-target RNAs are enriched for several biological functions, including those shown in Fig. 3J.

### UPF2 and UPF3B predominantly target different transcripts in human ESCs

**Figure 4.**
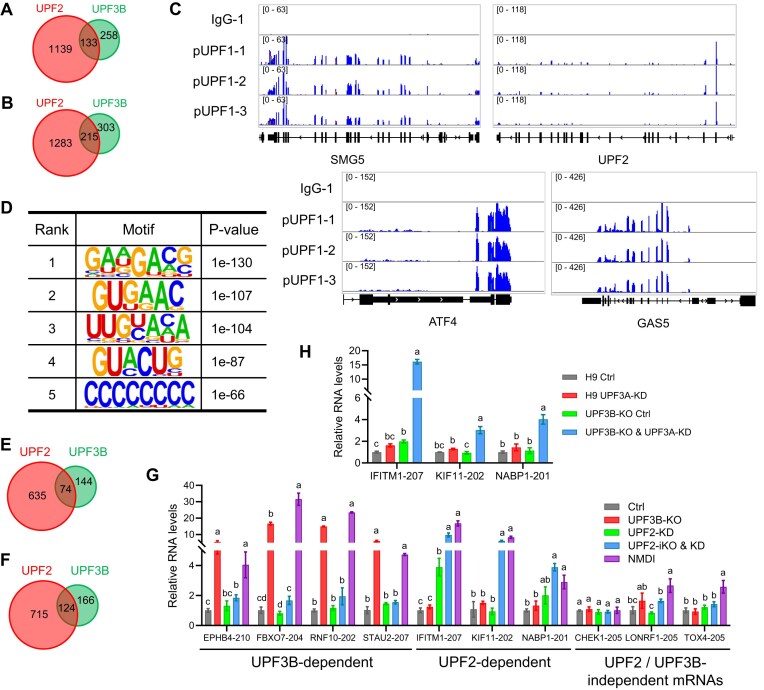
UPF2 and UPF3B predominantly target different transcripts in human ESCs. (**A**) Overlap of high-confidence UPF2- and UPF3B-dependent NMD-target RNAs (regulated by >2 fold, as defined in Figs 2 and 3, respectively). (**B**) Overlap of high-confidence UPF2- and UPF3B-dependent NMD-target RNAs exhibiting >1.5 fold regulation, following the same approaches as in Figs 2 and 3. (**C**) Integrative genomics viewer images showing tracks of normalized pUPF1 occupancy tag counts in ESCs analyzed by RIPseq. pUPF1-1, -2, and -3 are replicates from IP with a pUPF1 rabbit antisera. IgG-1 shows the signal when the IP was performed with normal rabbit antisera. (**D**) Sequence motifs enriched at pUPF1-occupied 3′ UTR sites, as determined using HOMER. (**E**) Overlap of UPF2- and UPF3B-dependent NMD-target RNAs regulated by >2-fold [defined in panel (A)] that exhibit pUPF1 occupancy in the 3′ UTR. (**F**) Overlap of UPF2- and UPF3B-dependent NMD-target RNAs regulated by >1.5-fold [defined in panel (B)] that exhibit pUPF1 occupancy in their 3′ UTR. (**G**) qPCR analysis of UPF2- and UPF3B-dependent NMD-target mRNAs in ESCs (defined by RNA-seq analysis) with the indicated genotypes and incubated with or without a UPF2 siRNA (UPF2-KD) (as in Fig. 1, above). Three non-NMD-target mRNAs (defined by RNA-seq analysis) were tested in parallel. Ctrl cells were also treated incubated with the small-molecule NMD inhibitor NMDI [113]. The ESC cells used and how they were cultured to deplete UPF2 are described in Fig. 1. Expression in nontreated Ctrl cells was set to “1.” The values shown are from two independently derived ESC clones, with two independent biological replicates. Statistical significance was determined using the Student’s *t*-test. Different letters (a, b, c, and d) denote statistically significant differences between different groups (*p* <0.05). (**H**) qPCR analysis of UPF2-dependent UPF3B-independent NMD-target mRNAs (as defined by RNA-seq analysis in ESCs). The expression of these NMD targets was evaluated in Ctrl or *UPF3B*-KO cells. *UPF3A*-KD refers to cells transfected with a *UPF3A*siRNA and cultured for 48 h before harvest. Both Ctrl and *UPF3B*-KO Ctrl cells were transfected with a scramble siRNA. The values shown are from two independently derived ESC clones, with two independent biological replicates. Statistical significance was determined using the Student’s *t*-test. Different letters (a, b, and c) denote statistically significant differences between different groups (*p* <0.05).

Our identification of UPF2- and UPF3B-dependent NMD mRNA targets in the same cells—human ESCs—provided an opportunity to directly compare the target specificity of UPF2 and UPF3B. Surprisingly, only 34% UPF3B-dependent NMD-target mRNAs (133/391) overlap with UPF2-dependent NMD-target mRNAs (Fig. 4A). This raised the possibility that instead of being an NMD-branch factor that only drives the decay of a subset of mRNAs degraded by the NMD pathway as a whole [9, 20–21, 49], UPF3B promotes the degradation of many unique targets that are UPF2-independent.

We considered the possibility that the somewhat stringent criterion used above to identify NMD targets (fold change >2) may exclude some genuine NMD-target mRNAs that exhibit only modest changes upon NMD factor depletion. This could affect our conclusions. To test this, we performed the same analyses as above using a less stringent cutoff (fold change >1.5). This cutoff criterion identified 1498 UPF2-dependent and 518 UPF3B-dependent high-confidence NMD-target transcripts (Supplementary Table S2). In support of UPF3B targeting many mRNAs for decay in a UPF2-indendent manner, only 41% of the UPF3B-dependent target mRNAs (215/518) overlap with UPF2-dependent target mRNAs, as defined using a >1.5-fold cutoff (Fig. 4B).

To increase confidence that *bona fide* NMD targets are being compared, we employed pUPF1 occupancy as “another” criterion to define direct NMD-target RNAs. It is well-established that UPF1 and pUPF1 persists on NMD-target mRNAs [83, 108, 109]. This assay relies on the evidence that the sequential phosphorylation and dephosphorylation of UPF1 is an integral part of NMD [83, 110].

To define mRNAs with high pUPF1 occupancy, we performed IP with a well-established antibody against pUPF1 [83] followed by RNA-seq (RIPseq) analysis. This RIPseq analysis identified 8980 genes encoding RNAs statistically significantly occupied by pUPF1 in human ESCs (based on peak signal value, with >1-fold enrichment above background; *q* <0.01; Supplementary Table S3). Since studies aiming to identify NMD targets have preferentially detected UPF1 in the 3′ UTR region [35, 83, 103, 111, 112], we focused our analysis on pUPF1 occupancy in the 3′ UTR region. pUPF1 occupancy of some known NMD-target mRNAs is shown in Fig. 4C. The top enriched motifs associated with pUPF1-occupied 3′ UTR sites are listed in Fig. 4D. This analysis revealed that 709/1272 (55%) and 218/391 (56%) of high-confidence UPF2- and UPF3B-dependent NMD targets, respectively, contain pUPF1 occupancy sites in their 3′ UTR (Supplementary Table S3).

Since pUPF1 occupancy is not known to mark all NMD targets, many of the above mRNAs that scored negative in this pUPF1 occupancy assay may also be NMD-target mRNAs. Nonetheless, to have confidence in our comparison of UPF2- and UPF3B-dependent targets, we compared only what we call “unambiguous” NMD-target mRNAs—those occupied by pUPF1. This analysis revealed that of the 709 and 218 unambiguous NMD targets dependent on UPF2 and UPF3B, respectively, only 74 overlap (Fig. 4E). To address the possibility that this small overlap pertains only to “strong” NMD-target RNAs, we also included less-regulated UPF2- and UPF3B-dependent NMD targets by using a fold change cutoff of >1.5, as described above (Fig. 4B). This analysis identified 839 and 290 NMD targets dependent on UPF2 and UPF3B, respectively, with only 124 overlapping (Fig. 4F).

For verification, we randomly selected UPF3B- and UPF2-specific NMD-target mRNAs identified by RNA-seq analysis, above, and examined their expression by qPCR analysis. In line with our RNA-seq analysis, all of the UPF3B-specific NMD-target mRNAs were upregulated in UPF3B-KO ESCs but not in UPF2-KD or -iKO & KD ESCs. Conversely, all of the UPF2-specific NMD-target mRNAs were upregulated in UPF2-KD or -iKO & KD ESCs but not in UPF3B-KO ESCs (Fig. 4G). The small-molecule NMD inhibitor, NMDI [113], upregulated all of these mRNAs (Fig. 4G), verifying that all these transcripts are NMD targets. For an additional level of verification, we used qPCR analysis to test the expression of three mRNAs that contain a dEJ, but are *not* regulated by either UPF2 or UPF3B, as defined by our RNA-seq analysis. qPCR analysis verified that none of these three mRNAs are significantly upregulated in UPF3B-KO, UPF2-KD, or UPF2-iKO & KD ESCs (Fig. 4G). Consistent with their containing a dEJ, two of these three mRNAs are NMD targets, based on their upregulation in response to NMDI (Fig. 4G).

Several studies have reported that UPF3B and its paralog, UPF3A, are functionally redundant [9, 44, 45], raising the possibility that some NMD targets we identified that are not affected by UPF3B KO might be upregulated in response to depletion of both UPPF3A and UPF3B. To test this, we selected three NMD-target RNAs that fulfilled three criteria: (i) upregulated in response to UPF2 depletion, (ii) pUPF1 occupancy in their 3′ UTR, and (iii) not upregulated in response to UPF3B KO. We found that all three of these mRNAs were upregulated in UPF3B-KO ESCs when UPF3A was also depleted (by RNAi; Fig. 4H). In contrast, UPF3A depletion alone did not significantly upregulate these three mRNAs (Fig. 4H). These results confirm that UPF3A and UPF3B can act redundantly.

The finding that UPF3B is essential for the decay of a large number of RNAs not targeted by UPF2 has implications for the underlying molecular mechanism of NMD, as well as for NMD’s roles in biology and disease. In the “Discussion” section, we discuss models to explain this surprising finding.

### Identification of NMD-target RNAs in human NPCs

It is typically assumed that the ability of an mRNA to be targeted for decay by NMD is mainly an intrinsic feature of the mRNA (see the “Introduction” section). However, to our knowledge, this assumption has remained largely unexplored. If correct, this assumption predicts that NMD-target mRNAs will be indifferent to cell type. To test this prediction, we compared UPF2-dependent NMD targets in human ESCs with those in human NPCs. To reduce variability, the NPCs we examined were derived from the same independent UPF2^fl/fl^ and UPF2^fl/fl^ Cre-ERT2 ESC clones (four ESC clones total) described above, following a standard protocol [60] (Supplementary Fig. S2A). Following generation of these NPCs, they were treated with 4-OHT and/or transfected with UPF2 siRNA to deplete UPF2 (Supplementary Fig. S3A). RNA-seq analysis was performed on these UPF2-deficient as well as Ctrl (UPF2^fl/fl^) human NPCs (3 experimental replicates from each clone, yielding 6 replicates per group; 24 samples total). Hierarchical clustering showed significant differences between the groups and little variation between replicates (Supplementary Fig. S3B). Through pairwise comparison with the Ctrl cells, we identified hundreds of dysregulated genes in the UPF2-KD, -iKO, and -iKO & KD groups (Supplementary Fig. S3C–E and Supplementary Table S4). Statistically significantly enriched functions associated with these dysregulated genes are shown in Supplementary Fig. S3C–E. Consistent with these UPF2-depleted NPCs exhibiting deficient NMD, these cells upregulated several well-established NMD-target mRNAs [8, 47, 54, 104, 106] (Supplementary Fig. S2B).

**Figure 5.**
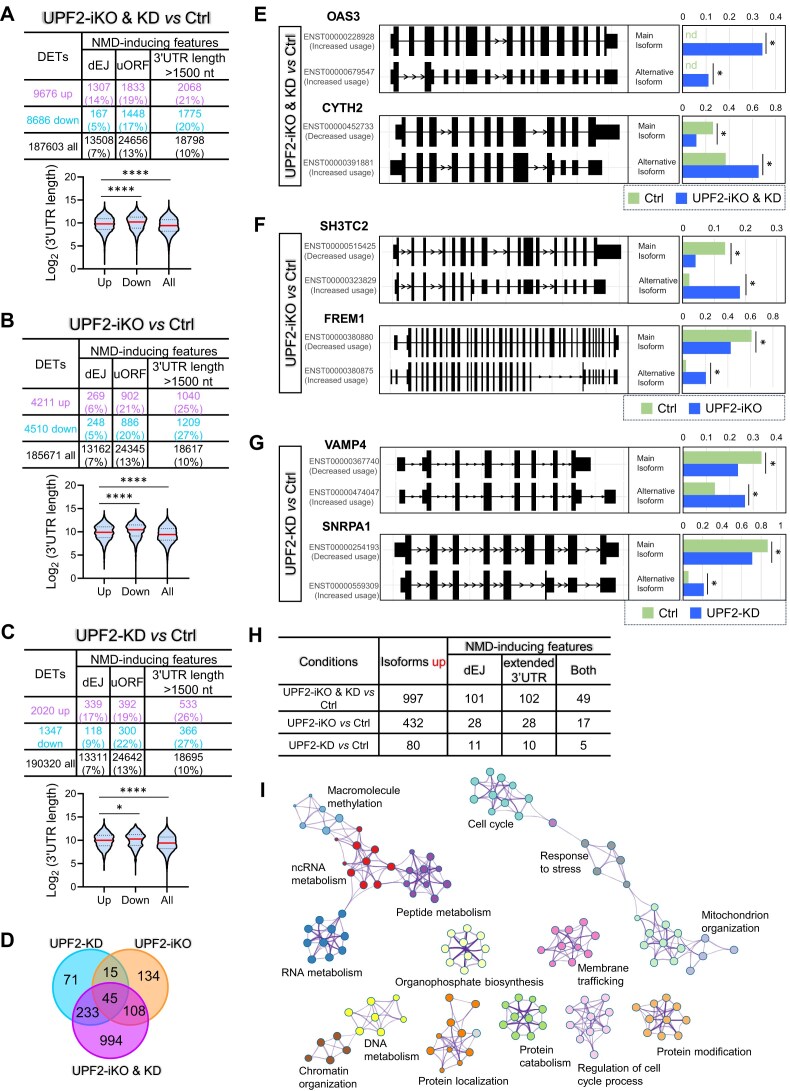
Identification of high-confidence NMD-target transcripts in human NPCs. (**A**–**C**) Top panel: The number of DETs, as well as the number of DETs with the indicated NMD-inducing feature, for the UPF2 disruption condition shown (determined from the datasets described in Supplementary Fig. S3). Bottom panel: Violin plot showing the 3′ UTR length of the up- and downregulated DETs, as well as all transcripts. * *p* <0.05; **** *p*<0.0001 (unpaired *t*-test). (**D**) Overlap amongst high-confidence NMD-target RNAs (those with a dEJ or an extended 3′ UTR) identified from the datasets in panels (A)–(C). (**E**–**G**) Examples of alternatively processed transcripts degraded by NMD in NPCs, as defined by the IsoformSwitchAnalyzeR program. The bar graph shows the expression of the main isoform versus an alternative isoform harboring an NMD-inducing feature that is upregulated by the indicated UPF2 disruption (other isoforms are not shown). Bonferroni-adjusted *t*-test * *q*<0.05; nd, nondetectable; ns, not statistically significant. (**H**) Alternatively processed mRNA isoforms upregulated by the indicated UPF2 perturbation condition, as defined by the IsoformSwitchAnalyzeR program. Shown is the total number of upregulated transcripts, as well as upregulated transcripts with the indicted NMD-inducing feature. “Both” refers to upregulated transcripts with both a dEJ and an extended 3′ UTR. (**I**) Biological functions defined by the Metascape program (*p*<0.01) associated with the full profile of high-confidence NMD-target mRNAs in ESCs [defined from the datasets in panels (A)–(C) and (H)].

We identified high-confidence NMD-target mRNAs in these human NPCs using the same two approaches, described above, for human ESCs. The RSEM program [73] identified 18 362, 8721, and 3367 statistically dysregulated transcripts in the UPF2-iKO & KD, -iKO, and -KD groups, respectively (Fig. 5A–C and Supplementary Table S4). Consistent with our finding from human ESCs, dEJs are enriched in the transcripts upregulated in response to UPF2-iKO & KD or UPF2-KD (Fig. 5A and C). For example, 14% of UPF2-iKO & KD-upregulated transcripts in human NPCs harbored a dEJ, whereas only 5% of transcripts downregulated by the same condition harbored a dEJ (Fig. 5A). In contrast, the UPF2-iKO condition did not elicit this enrichment (Fig. 5B), just as we observed for human ESCs (Fig. 2B), providing more evidence that the UPF2-iKO condition is unique in capturing non-dEJ-enriched NMD targets. Together, the analyses portrayed in Fig. 5A–C identified 1499 dEJ-containing mRNAs that are negatively regulated by UPF2 in human NPCs and thus likely direct NMD-target mRNAs in human NPCs.

In contrast with dEJs, neither uORFs nor long 3′ UTRs (>1500 nt) are enriched in upregulated transcripts in UPF2-depleted human NPCs (Fig. 5A–C). Furthermore, the average length of the 3′ UTR is not significantly longer (instead it is significantly shorter) in upregulated transcript versus downregulated transcripts in response to UPF2-iKO & KD or UPF2-iKO conditions; there is also no significant difference in this length for the UPF2-KD condition (Fig. 5A–C). While these results indicate that 3′ UTR length is not a robust feature to predict NMD targets in human NPCs, it does not rule out that long 3′ UTRs can engage NMD to degrade some transcripts in these cells. Indeed, we identified 218, 73, and 70 upregulated transcripts having extended 3′ UTR relative to the main isoform in the UPF2-iKO&KD, UPF2-iKO, and UPF2-KD conditions, respectively (Supplementary Table S4). As with ESCs, the majority of these alternatively processed transcripts with an extended 3′ UTR in NPCs also have a dEJ (145/218, 40/73, and 45/70 transcripts upregulated in the UPF2-iKO & KD, UPF2-iKO, and UPF2-KD conditions, respectively). In total, RSEM-based analysis identified a total of 1600 high-confidence NMD-target transcripts that are upregulated in human NPCs in response to UPF2 perturbation and have a dEJ and/or an extended 3′ UTR (Fig. 5D).

Using the IsoformSwitchAnalyzeR pipeline [75], we identified 1119, 547, and 110 genes expressing transcripts undergoing isoform switches in response to the UPF2-iKO & KD, UPF2-iKO, and UPF2-KD conditions, respectively (Fig. 5E–G and Supplementary Table S4). Of these genes, the majority (899, 400, and 78 genes, respectively) give rise to upregulated mRNA isoforms: 997, 432, and 80 transcripts, respectively (Supplementary Table S4). Fig. 5H indicates the proportion of these transcripts that had a dEJ and/or an extended 3′ UTR. The percentage of upregulated transcripts with an extended 3′ UTR is modestly higher for the UPF2-KD condition (12.5%; 10/80) than for the UPF2-iKO & KD (10.2%; 102/997) and the UPF2-iKO conditions (6.5%; 28/432), suggesting that this particular condition enriches for this NMD-inducing feature in human NPCs. Fig. 5E–G provide examples of the types of alternatively processing undergone by the upregulated isoforms versus the main isoform, as well as their degree of regulation. The total number of upregulated alternatively processed transcripts with a dEJ and/or extended 3′ UTR identified by the IsoformSwitchAnalyzeR pipeline is 178; 75 of these overlap with those identified using the RSEM program. Thus, the combination of both approaches allowed us to identify 1703 (1600 + 178 − 75) high-confidence NMD-target RNAs in human NPCs. These 1703 transcripts are statistically enriched for several biological functions (Fig. 5I).

### Cell type-specific NMD-target mRNAs are the norm rather than the exception

**Figure 6.**
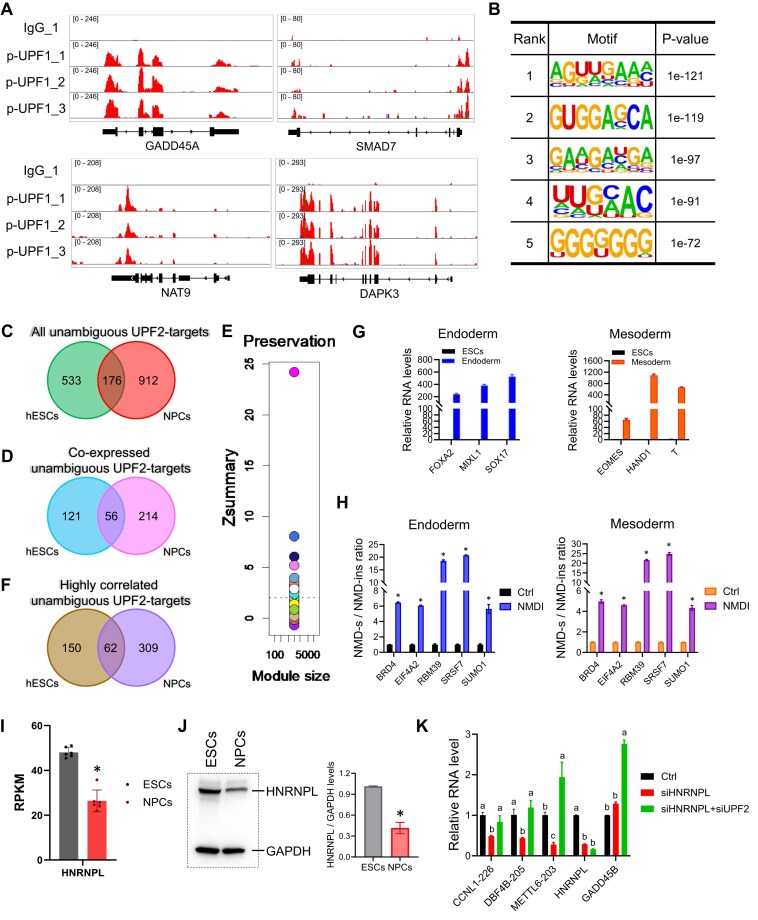
Most NMD-target mRNAs are cell type-specific. (**A**) Integrative genomics viewer images showing tracks of normalized pUPF1 occupancy tag counts in human NPCs. Three biological replicates, along with one negative Ctrl (IgG), are shown. (**B**) Sequence motifs enriched at pUPF1-occupancy sites, as determined using HOMER. (**C**) Overlap of all unambiguous UPF2-dependent NMD-targeted RNAs in human ESCs and NPCs. (**D**) Overlap of only unambiguous UPF2-dependent NMD-targeted RNAs expressed in both human ESCs and NPCs (expression cutoff: >5 TPM). (**E**) Identification of transcripts co-expressed in human ESCs and NPCs as defined by module preservation analysis using the WGCNA program (Zsummary >2 was used as the cutoff). Different colors of the dots represent individual modules with different Zsummary statistic. *X*-axis, gene number in each module. (**F**) Overlap of only unambiguous UPF2-dependent NMD-target RNAs co-expressed in both ESCs and NPCs as defined by the module preservation analysis in panel (E). (**G**) qPCR analysis of human ESCs differentiated into endoderm and mesoderm, respectively, using standard protocols [59]. Shown are well-established markers for the indicated germ layers. Data are represented as mean ± SEM (n = 3). (**H**) NMD-sensitive/NMD-insensitive mRNA isoform ratio (determined by qPCR analysis) of the indicated genes in endoderm and mesoderm treated with the small-molecule NMD inhibitor NMDI [113] or the vehicle, dimethyl sulfoxide (DMSO), alone (Ctrl). The NMD-sensitive mRNA isoform from each of these five genes is an NMD target in ESCs and NPCs (Supplementary Table S5). The expression of the individual NMD-sensitive and -insensitive transcripts is shown in Supplementary Fig. S5. Statistical significance was determined using the Student’s *t*-test. *n* = 3; * *p* <0.05. (**I**) HNRNPL expression in human ESCs versus NPCs, as detected by RNA-seq. (**J**) Western blot analysis of HNRNPL protein levels in human ESCs versus NPCs. The right panel shows mean HNRNPL levels relative to the internal Ctrl (GAPDH; *n* = 3). (**K**) qPCR analysis of human ESCs transfected with siRNAs against HNRNPL or UPF2. *CCNL1-226*, *DBF4B-205*, and *METTL6-203* are NMD-target RNAs in NPCs, not ESCs. These transcripts have 70, 55, and 88 predicted HNRNPL-binding sites, respectively, based on the RBPmap program [126]. *GADD45B* is a negative-Ctrl NMD-target mRNA [36] with only four predicted HNRNPL-binding sites. Statistical significance was determined using the Student’s *t*-test (*n* = 3). Different letters (a, b, and c) denote statistically significant differences between different groups (*p* < 0.05).

Having defined high-confidence NMD targets in both human ESCs and NPCs, we were in a position to determine whether most NMD targets are shared or unique. To be stringent, we defined unambiguous NMD-target RNAs in these two cell types by employing pUPF1 occupancy (see above) as another criterion. Fig. 4C and D and Supplementary Table S3 provide information on pUPF1 occupancy sites in human ESCs. Fig. 6A and B and Supplementary Table S5 provide information on pUPF1 occupancy sites in human NPCs. Together, these analyses identified a total of 709 and 1088 NMD-target transcripts occupied by pUPF1 in ESCs and NPCs, respectively. All these unambiguous NMD-target RNAs fulfill three criteria of being direct NMD targets: (i) upregulation in response to UPF2 perturbation, (ii) possession of an NMD-inducing feature (a dEJ and/or an extended 3′ UTR), and (iii) 3′ UTR pUPF1 occupancy.

Overlap analysis revealed that only a small proportion of these unambiguous NMD-target mRNAs are degraded by NMD in both ESCs and NPCs (Fig. 6C). Only 25% (176/709) of NMD targets in ESCs are also NMD targets in NPCs; and only 16% (176/1088) of NMD targets in NPCs are also NMD targets in ESCs. To test the possibility that this low overlap is because weakly responsive NMD-target mRNAs were excluded by the >2 fold regulation cutoff we used above, we used a >1.5-fold cutoff (Supplementary Tables S1 and S4). This identified 839 and 1221 NMD-target transcripts occupied by pUPF1 in ESCs and NPCs, respectively. Overlap analysis showed that only 26% (219/839) of NMD-target RNAs in ESCs are also NMD-target RNAs in NPCs; and only 18% (219/1221) of NMD-target mRNAs in NPCs are also NMD-target RNAs in ESCs.

One intriguing explanation for this finding is these two cell types differ fundamentally in what they consider to be an NMD target. However, an alternative possibility is that this cell type specificity is merely because most NPC-specific NMD targets are not expressed in ESCs, and, conversely, most ESC-specific NMD targets are not expressed in NPCs. We used two approaches to distinguish between these two possibilities. First, we did overlap analysis of only transcripts co-expressed in these two cell types. Using TPM >5 as a stringent cutoff (to restrict analysis to relatively highly-expressed mRNAs), we found that 177 NMD-target RNAs expressed in ESCs are co-expressed in NPCs, and 270 NMD-target RNAs expressed in NPCs are co-expressed RNAs in ESCs (Fig. 6D). Of these co-expressed NMD-target mRNAs, only 56 overlap between ESCs and NPCs (Fig. 6D). As a second approach, we applied module preservation analysis using the WGCNA framework [82], which places genes with highly correlated expression patterns into individual modules or clusters. Through this module preservation test, we identified 38 262 transcripts that exhibit highly correlated expression patterns in ESCs and NPCs (Zsummary score >2; Fig. 6E). Of these 38 262 WGCNA module-defined transcripts, 212 and 371 are NMD-target RNAs in ESCs and NPCs, respectively (Fig. 6F). Only 62 of these WGCNA module-defined RNAs overlap between ESCs and NPCs (Fig. 6F). Supplementary Fig. S4A–C show Sashimi plots of examples of ESC- and NPC-specific NMD target isoforms, as well as “common” NMD target isoforms. Together, these results strongly suggest that ESCs and NPCs intrinsically differ in their decision as to what they regard is an NMD-target mRNA. These results support a model that posits the existence of a developmental switch that triggers a large class of mRNAs to become sensitized to NMD when ESCs progress to form NPCs. Another large class of mRNAs escape from NMD during this transition. Surprisingly, the majority of NMD-target mRNAs are in either of these two classes; with only a minority being susceptible to NMD in both ESCs and NPCs.

### NMD reporter transcripts

The 62 transcripts targeted by NMD in both ESCs and NPCs (described above; Fig. 6F) have potential utility as reporters of NMD magnitude in a variety of cell types. As support, some of the genes transcribing these mRNAs—including *SRSF7*, *EIF4A2*, and *RBM39*—have been previously shown to encode NMD-target mRNAs in other cell types [114–116]. To provide a resource for predicting NMD magnitude, we summarize these 62 unambiguous NMD-target RNAs in Supplementary Table S5. This table also lists NMD-insensitive RNA isoforms from the same genes; these can be analyzed in parallel to correct for changes in transcription. As a validation, we selected 5 of these 62 NMD-target transcripts to determine whether they are also targeted for decay by NMD in endoderm and mesoderm, two of the three germ layers differentiated from ESCs (the third being neuroectoderm, which gives rise to NPCs). To address this question, we generated endoderm and mesoderm cells that express high levels of markers specific for endoderm and mesoderm, respectively (Fig. 6G). Treatment of these endoderm and mesoderm cells with the small-molecule NMD inhibitor, NMDI [113], upregulated the NMD-sensitive mRNA isoform transcribed from all five of these genes (Supplementary Fig. S5). In contrast, the NMD-insensitive mRNA isoform from these same genes exhibited either no significant change in expression or only a modest effect (Supplementary Fig. S5), which is accounted for by displaying the data as NMD-sensitive/NMD-insensitive ratio (Fig. 6H). This set of NMD-sensitive/NMD-insensitive transcripts from the *BRD4*, *EIF4A2*, *RBM39*, *SRSF7*, and *SUMO1* genes can potentially be used as an unambiguous NMD reporter (impervious to transcriptional alterations) in a variety of cell types.

### HNRNPL mediates cell type-specific regulation of a subset of NMD-target mRNAs

It has been shown that transcripts can escape NMD if occupied in their 3′ UTR by the RNA-binding protein HNRNPL [36]. This led us to hypothesize that the differential occupancy of HNRNPL has a role in cell type-specific NMD. In particular, since HNRNPL expression is higher in ESCs than NPCs (Fig. 6I and J), we hypothesized that NMD-target transcripts with high HNRNPL occupancy escape NMD in ESCs due to this HNRNPL-dependent mechanism. To test this hypothesis, we randomly selected three NMD-target RNAs with a high number of HNRNPL occupancy sites in their 3′ UTR regions that are NMD targets in NPCs but not ESCs. In response to HNRNPL depletion, all three of these NMD-target RNAs are downregulated in ESCs, consistent with HNRNPL protecting them from decay by NMD (Fig. 6K). As direct support for this downregulatory response being caused by NMD, it was eliminated when UPF2 was knocked down in parallel (Fig. 6K). As a negative Ctrl, we tested *GADD45B* mRNA, a well-established NMD-target mRNA with few HNRNPL binding sites that was previously shown to not respond to HNRNPL KD [36]. *GADD45B* mRNA levels did not significantly change in response to HNRNPL depletion in ESCs (Fig. 6K).

### Identification of UPF3B-dependent NMD-target RNAs in human NPCs

**Figure 7.**
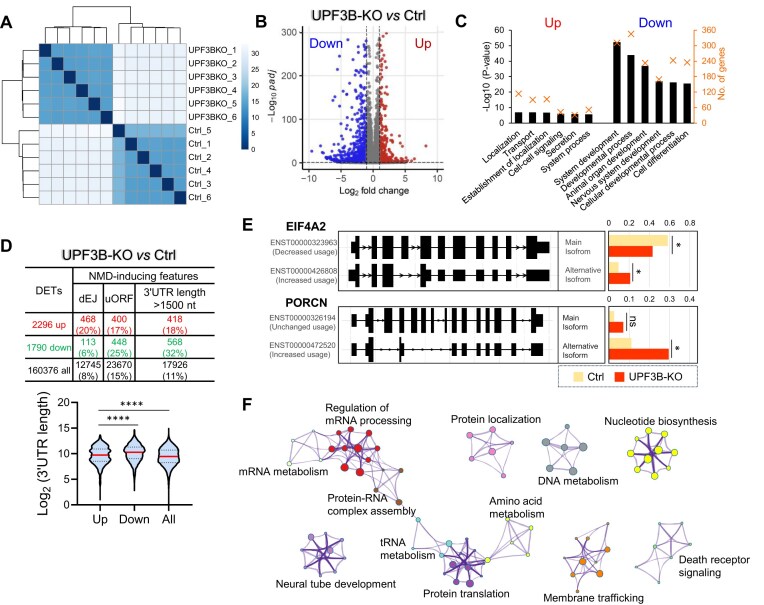
Identification of high-confidence UPF3B-dependent NMD-target RNAs in human NPCs. (**A**) Unsupervised hierarchical clustering of two independent clones of Ctrl and *UPF3B*-KO human NPCs assayed by RNA-seq analysis (three experimental replicates for each group). (**B**) DEGs (*q* <0.01, fold change >2) identified from the RNA-seq analysis in panel (A). (**C**) Biological functions associated with the DEGs defined in panel (B). Statistical significance [−Log_10_ (*p*-value)] is indicated by the bar. The number of DEGs for a given category is indicated by an “X.” (**D**) Top panel: The number of DETs, as well as the number of DETs with the indicated NMD-inducing feature. Bottom panel: Violin plot showing the 3′ UTR length of the up- and down-regulated DETs, as well as all transcripts. **** *p* <0.0001 (unpaired t-test). (**E**) Examples of alternatively processed transcripts degraded by UPF3B-dependent NMD in NPCs, as defined by the IsoformSwitchAnalyzeR program. The bar graph shows the expression of the main isoform versus an alternative isoform harboring an NMD-inducing feature that is upregulated by UPF3B loss (other isoforms are not shown). Bonferroni-adjusted *t*-test * *q*<0.05; ns, not statistically significant. (**F**) Biological functions defined by the Metascape program (*p*<0.01) associated with the full profile of high-confidence UPF3B-dependent NMD-target transcripts in NPCs.

To determine the generality of cell type-specific NMD, we extended our analysis to UPF3B-dependent NMD-target mRNAs. This required that we define such target mRNAs in humans NPCs. To achieve this, we derived NPCs from human *UPF3B*-KO ESCs and then performed the same RNA-seq and downstream analyses as described above. Hierarchical clustering analysis of the six biological replicates of the *UPF3B*-KO and Ctrl NPCs showed the expected clustering (Fig. 7A). A total of 1107 genes were dysregulated in the *UPF3B*-KO NPCs, with 469 undergoing upregulation and 638 undergoing downregulation (Fig. 7B and Supplementary Table S6). Statistically enriched functions associated with these upregulated and downregulated genes are shown in Fig. 7C. Several well-established NMD targets were upregulated in these *UPF3B*-KO NPCs (Supplementary Fig. S2C), consistent with these cells exhibiting impaired NMD.

We next identified UPF3B-target RNAs at the transcript level, following the same approaches as described above. Using the RSEM program [73], we identified 2296 transcripts that are upregulated in the *UPF3B*-KO NPCs (FDR <0.01, |log_2_FC| >1; Supplementary Table S6). dEJs are highly enriched in these upregulated transcripts (20% dEJ+ versus 6% dEJ+ downregulated transcripts; Fig. 7D). In contrast, neither uORFs nor long 3′ UTRs are enriched in the upregulated transcripts (Fig. 7D), consistent with what we observed for upregulated transcripts in *UPF3B*-KO ESCs (Fig. 3H). The average length of the 3′ UTR is significantly shorter in upregulated transcript versus downregulated transcripts (Fig. 7D). To identify candidates that are downregulated by NMD through long 3′ UTRs, we used the same approach as described above, which identified 99 transcripts having extended 3′ UTR relative to the main isoform (Supplementary Table S6). A large proportion of these transcripts also have a dEJ (69/99). In total, RSEM analysis identified 498 high-confidence UPF3B-dependent NMD-target mRNAs that are upregulated in response to UPF3B loss and have a dEJ and/or an extended 3′ UTR.

To identify more alternatively processed transcripts degraded by UPF3B-dependent NMD, we used the IsoformSwitchAnalyzeR pipeline [75]. This pipeline identified 83 genes encoding 133 RNA isoforms undergoing a significant alteration in isoform usage in UPF3B-KO versus Ctrl NPCs (*q*<0.01) (Supplementary Table S6). Focusing on the 71 upregulated isoforms (derived from 68 distinct genes), 12 have a dEJ and 8 had an extended 3′ UTR relative to the main isoform (Fig. 7E and Supplementary Table S6). In total, 13 upregulated transcripts with a dEJ and/or extended 3′ UTR were defined.

The total number of upregulated alternatively processed transcripts with a dEJ and/or extended 3′ UTR identified by the above two approaches is 503. These high-confidence UPF3B-dependent NMD-target RNAs in NPCs are enriched for several biological functions, including “neural tube development” (Fig. 7F).

### NMD-target mRNAs are largely NMD factor- and cell type-specific

**Figure 8.**
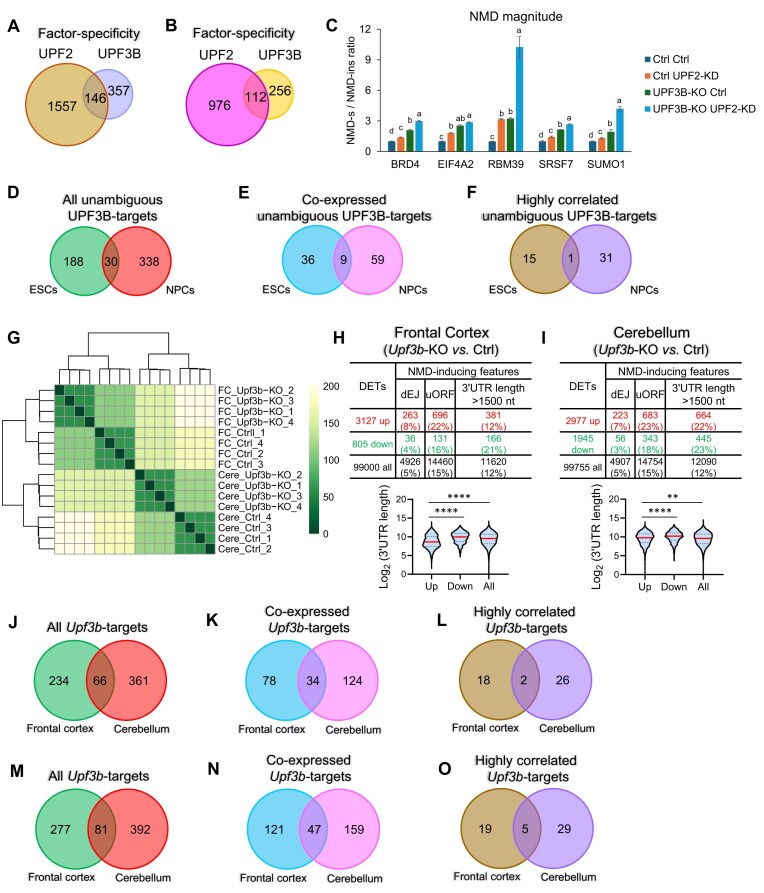
NMD factor-, cell type-, and tissue-specific NMD. (**A**) Overlap of all high-confidence UPF2- and UPF3B-dependent NMD-target RNAs in NPCs (defined in Figs 5 and 7, respectively). (**B**) Overlap of all unambiguous UPF2- and UPF3B-dependent NMD-target RNAs [those in panel (A) that are occupied by pUPF1]. (**C**) Effect of combined UPF3B loss and UPF2 KD on NMD magnitude in NPCs, as quantified by NMD-sensitive/NMD-insensitive mRNA isoform ratio (see Fig. 6H). Expression of the mRNA isoforms was determined by qPCR analysis of NPCs with the indicated genotypes (Ctrl or *UPF3B*-KO) transiently transfected with a *UPF2* siRNA or a negative control scramble siRNA (Ctrl). The expression of the individual NMD-sensitive and -insensitive transcripts is shown in Supplementary Fig. S6. The values shown are from two independently derived UPF3B-KO clones, with two independent biological replicates. Statistical significance was determined using the Student’s *t*-test. Different letters (a, b, c, and d) denote statistically significant differences between different groups (*p* <0.05). (**D**) Overlap of all unambiguous UPF3B-dependent NMD-target RNAs expressed in human ESCs and NPCs. (**E**) Overlap of only unambiguous UPF3B-dependent NMD-target RNAs co-expressed in both ESCs and NPCs (cutoff: >5 TPM). (**F**) Overlap of only unambiguous UPF3B-dependent NMD-target RNAs co-expressed in both ESCs and NPCs as defined by module preservation analysis. (**G**) Unsupervised hierarchical clustering of frontal cortex and cerebellum from four adult *Upf3b*-null and four littermate control (Ctrl) mice analyzed by RNA-seq analysis. (**H** and **I**) Top panel: The number of DETs, as well as the number of DETs with the indicated NMD-inducing feature. Bottom panel: Violin plot showing the 3′ UTR length of the up- and down-regulated DETs, as well as all transcripts. ** *p* <0.01; **** *p* <0.0001 (unpaired t-test). (**J**) Overlap of all high-confidence UPF3B-dependent NMD-target RNAs (those with a dEJ and/or an extended 3′ UTR) that are expressed in mouse frontal cortex or cerebellum [defined from the datasets shown in panels (H) and (I), respectively]. (**K**) Overlap of only high-confidence UPF3B-dependent NMD-target RNAs co-expressed in both mouse frontal cortex and cerebellum (cutoff: >5 TPM). (**L**) Overlap of only high-confidence UPF3B-dependent NMD-target RNAs co-expressed in both mouse frontal cortex and cerebellum as defined by module preservation analysis. (**M**–**O**) Overlap analyses performed the same as in panels (J)–(L) except that the fold change cutoff used to define UPF3B-dependent NMD-target RNAs in mouse frontal cortex or cerebellum was >1.5 rather than >2.

Having defined UPF3B-dependent NMD-target mRNAs in NPCs (Fig. 7), we were in a position to ask three questions:

First, in NPCs, does UPF3B mainly target (i) a subset of UPF2-dependent mRNA targets (as predicted by the dogma in the field) or (ii) different mRNAs than UPF2 (which would imply different mechanisms of action for these two NMD factors)? In support of outcome (ii), we found that only 29% (146 of 503) UPF3B-dependent target RNAs overlap with UPF2-dependent target RNAs in NPCs (Fig. 8A). To test the possibility that this low overlap is because weakly responsive NMD-target mRNAs were excluded by the >2 fold regulation criteria we used above, we also used a >1.5 fold regulation cutoff. This identified 1886 UPF2- and 606 UPF3B-dependent target mRNAs in NPCs, respectively (Supplementary Tables S4 and S6). Overlap analysis showed that only 31% (190/606) UPF3B-dependent target RNAs overlap with UPF2-dependent target RNAs in NPCs, as defined using a >1.5-fold cutoff. The low overlap between UPF2- and UPF3B-dependent NMD targets in NPCs (observed with either a >1.5- or >2-fold cutoff) is consistent with what we observed in ESCs (Fig. 4A and B).

To more stringently assess whether UPF2 and UPF3B largely target different transcripts for decay in NPCs, we used pUPF1 occupancy as another NMD target criteria, determined as described above. This analysis defined 1088 and 368 unambiguous NMD targets dependent on UPF2 and UPF3B, respectively, in NPCs (Supplementary Tables S5 and S6). Only 30% (112 of 368) of these unambiguous UPF3B-dependent targets overlap with the unambiguous UPF2-dependent targets in NPCs (Fig. 8B), similar to the 34% overlap of unambiguous targets observed in ESCs (Fig. 4E). Together, this provides strong evidence that UPF2 and UPF3B predominantly target different transcripts for decay.

Second, do UPF3B and UPF2 cooperate on the target mRNAs they have in common? We addressed this question using the five “NMD reporter” transcripts described above in Fig. 6H. All five of these NMD-target transcripts were upregulated by either UPF2 depletion or UPF3B loss (Fig. 8C and Supplementary Fig. S6). A combination of UPF3B loss and UPF2 depletion resulted in either an additive or synergistic increase in the level of these NMD-sensitive transcripts (Fig. 8C and Supplementary Fig. S6). Thus, even though UPF3B and UPF2 largely target different transcripts for decay, they cooperate on those few targets they have in common. This is consistent with the considerable evidence that UPF3B and UPF2 exert different biochemical functions in the NMD pathway [3–6, 10].

Third, does UPF3B act in a cell type-specific manner? As described above, we found that UPF2 primarily targets different transcripts for decay in ESCs versus NPCs (Fig. 6). We asked if this was also the case for UPF3B. Comparison of the >210 UPF3B-dependent NMD targets in NPCs (Fig. 7) and ESCs (Fig. 3) revealed that only 30 target RNAs overlap in these two cell types (Fig. 8D). When examining only co-expressed mRNAs (by using a stringent TPM value of >5 as a cutoff to focus on relatively highly expressed mRNAs), only nine RNAs overlap (Fig. 8E). Finally, when using WGCNA program to define mRNAs with correlated expression patterns, only one RNA overlaps (Fig. 8F). Together, these analyses provide compelling evidence that UPF3B-dependent NMD-target mRNAs, like UPF2-dependent NMD-target mRNAs, are largely cell type-specific.

### Tissue-specific NMD-target transcripts *in vivo*

To assess generality, we examined whether NMD tends to target different mRNAs for decay in different regions of the same organ *in vivo*. We chose to examine frontal cortex and cerebellum, which are composed of related cells (neurons and glial cells) but differ in their nervous system functions. We defined UPF3B-dependent target mRNAs in these 2 brain regions using *Upf3b*-KO mice, which possess highly specific behavioral defects but otherwise appear normal, including having normal fertility [21]. We performed RNA-seq analysis on frontal cortex and cerebellum from four adult *Upf3b*-null and four littermate Ctrl mice (Fig. 8G). Using the same RSEM and IsoformSwitchAnalyzeR pipelines described above, we identified 300 and 427 high-confidence UPF3B-dependent target RNAs that contain a dEJ and/or an extended 3′ UTR in frontal cortex and cerebellum, respectively (Fig. 8H and I and Supplementary Table S6). Only 66 of these NMD targets overlap between frontal cortex and cerebellum (Fig. 8J). Even when considering only co-expressed mRNAs (using a TPM >5 cutoff), only 34 NMD targets overlap (Fig. 8K). Finally, when considering only mRNAs with correlated expression patterns (as defined using the WGCNA program described above), only two NMD targets overlap (Fig. 8L).

To test the possibility that this low overlap between NMD targets in frontal cortex and cerebellum is because weakly responsive NMD-target mRNAs were excluded by the >2-fold cutoff we used above, we also used a >1.5-fold cutoff. This identified 358 and 473 high-confidence UPF3B-dependent target RNAs in frontal cortex and cerebellum, respectively (Supplementary Table S6). Only 81 of these NMD targets overlap between frontal cortex and cerebellum (Fig. 8M). When considering only co-expressed mRNAs (using a TPM >5 cutoff), only 47 NMD targets overlap (Fig. 8N). Finally, when considering only mRNAs with correlated expression patterns (as defined using the WGCNA program described above), only five NMD targets overlap (Fig. 8O). Together, these results suggest that UPF3B predominately targets different mRNAs for decay in mouse frontal cortex versus cerebellum.

## Discussion

The notion that NMD-target mRNAs are a specialized group of mRNAs is widely accepted. In large part, this comes from the discovery of specific *cis-*elements (or “features”) in mRNAs that trigger their decay by the NMD pathway [3–6, 18, 40, 41]. Here, we determined whether cell type also has an influence on the decision as to whether or not an mRNA is degraded by NMD. Given the wealth of evidence that specific mRNA features trigger RNA decay through NMD, we anticipated most NMD-target mRNAs would be insensitive to cell context. Thus, we were surprised to find that the vast majority of NMD-target mRNAs in human ESCs differ from those in human NPCs (Figs 6 and 8). Given this unexpected result, we considered the possibility that our criteria for defining NMD-target mRNAs was insufficient, so we also used another criterion—pUPF1 occupancy—and obtained the same result (Figs 4, 6, and 8). We also considered the possibility that ESCs and NPCs express different NMD-target mRNAs simply because their transcriptomes differ. To address this, we focused our analysis only on mRNAs co-expressed in ESCs and NPCs (using two different approaches) and found, again, that most NMD targets in ESCs are not NMD targets in NPCs, and vice versa (Figs 6 and 8). We also assessed whether this cell type-specific response was a peculiarity of NMD-target mRNAs that depend on a specific NMD factor. Contrary to this hypothesis, we found that both UPF2- and UPF3B-dependent NMD targets were predominantly cell type-specific (Figs 6 and 8). To determine whether cell context-dependent NMD is merely an *in vitro* phenomenon, we also asked if it also occurs *in vivo*. We compared two different brain regions in adult mice—frontal cortex and cerebellum—and found that there was only modest overlap in their UPF3B-dependent NMD-target mRNAs (Fig. 8). Together, these several lines of evidence strongly support the notion that cellular context is a major determinant as to whether or not an mRNA is degraded by the NMD pathway.

A large variety of functions have been ascribed to the NMD pathway [3–6, 18, 40, 41]. Molecular mechanisms by which NMD impacts some of these functions have begun to be ascertained. For example, rescue experiments have shown that NMD degrades *c-Myc*, *Eif4a2*, *Gadd45*, *IRE1α*, and *SMAD7* mRNA to influence stem cell differentiation, pluripotency, programmed cell death, the unfolded protein response, and neural differentiation, respectively [19, 23, 48, 90, 115, 117]. While these findings are significant, it remains unclear what other mRNAs are degraded by NMD to influence these biological events. Furthermore, the NMD-target mRNAs responsible for NMD’s many other reported functions are also unknown. Our study herein strongly suggests that the list of NMD-target mRNAs that need to be considered as candidates that mediate a given NMD function will need to match the cell type under consideration. For example, the list of NMD-target mRNAs we identified in ESCs provides candidates to be responsible for NMD’s ability to influence human ESC fate [59] and possibly NMD’s essential roles in early embryonic development [22, 24]. Likewise, the list of NMD-target mRNAs we identified in NPCs are candidates to act downstream of NMD to influence neural differentiation [20, 21, 48, 51, 118]. In contrast, these lists of NMD-target mRNAs will probably be of little use in determining how NMD influences differentiation and developmental events in other cell types. This even applies to ubiquitously expressed mRNAs, as we found that most NMD targets are cell type-specific, even when only considering broadly expressed mRNAs. Thus, to determine the molecular mechanism of action of NMD in a given cell type, it will probably be necessary to define the full-spectrum of NMD targets in that particular cell type. The same applies to determining how NMD influences genetic diseases. B-lymphoblastoid cell lines from disease patients are often used to model their disease, including analysis of their profile of NMD-target mRNAs [32, 52, 53], but our results raise the possibility that most of the NMD-target mRNAs defined in B-lymphoblastoid cells will not be NMD targets in the cells responsible for the disease.

It will be interesting to determine whether there are patterns underlying which RNAs are targeted by NMD in different cell types. For example, do cells of a given lineage tend to have related NMD-target specificity? We found that NMD targets in brain frontal cortex and cerebellum are largely different (Fig. 8), which raises the possibility that even related cell types have vastly different NMD targets. But it is also possible that the large difference we observed is the result of a single neural cell type with a unique NMD target spectrum that is differentially present in frontal cortex versus cerebellum.

Another interesting future question is how NMD-target specificity is altered in a given lineage of cells as they progress from stem cells to progenitors and then undergo differentiation. Our results showed that hundreds of mRNAs become either sensitized or desensitized to NMD as ESCs transition to form NPCs (Fig. 6), suggesting the existence of a major NMD switch operating during this developmental transition. It is possible that such NMD developmental switches occur in other cell lineages, as a previous analysis of a special class of NMD-target mRNAs (those that encode NMD factors) showed that these mRNAs are differentially targeted by NMD in different tissues and cell types [54]. NMD sensitivity switching could have a profound impact on developmental events. For example, loss of NMD sensitivity could be a mechanism to induce mRNAs during development and thereby elicit new developmental programs. Conversely, acquisition of NMD sensitivity could be a mechanism to downregulate mRNAs involved in the earlier stage of development.

What molecular mechanisms underly cell type-specific NMD? We provide evidence that one mechanism involves HNRNPL, a RNA-binding protein that was previously shown to drive NMD escape when bound to the 3′ UTR of an mRNA [36]. We found that HNRNPL is differentially expressed in human ESCs versus NPCs in a manner that protects a subset of NMD-target mRNAs from NMD in ESCs, not NPCs (Fig. 6). Genome-wide analysis of a variety of cell types that express different levels of HNRNPL will be required to determine the full extent of HNRNPL’s role in cell type-specific NMD. It will also be important to determine whether there are other RNA-binding proteins that confer cell type-specific NMD through such an NMD escape mechanism. A candidate is PTBP1, since, like HNRNPL, it binds to the 3′ UTR of transcripts to inhibit UPF1 recruitment and thereby drive NMD escape [37]. It is also possible that some RNA-binding proteins confer cell type-specific NMD by promoting NMD rather than driving NMD escape. Selectivity would be conferred by the cell type-specific expression of these NMD-promoting RNA-binding proteins or the presence of absence of co-factors that work with these RNA-binding proteins in specific cell types. The regulation conferred by such RNA-binding proteins, regardless of whether they stimulate or suppress NMD, could also have a role in the variation in NMD-target mRNA levels recently observed in otherwise homogeneous cell populations [119].

Our study also addressed another dogma in the field—that NMD is driven by two kinds of proteins: core factors that promote the decay of most NMD-target mRNAs and branch-specific factors required for the decay of only subsets of these NMD-target mRNAs. In this report, we focused on a putative NMD-branch factor of considerable interest—UPF3B. As described in the “Introduction” section, UPF3B has key roles in the nervous system. For example, UPF3B is critical for neural differentiation *in vitro* and dendritic spine maturation *in vivo* [21, 51, 58]. *UPF3B* gene mutations are known to cause intellectual disability and are associated with neuropsychiatric disorders, including schizophrenia, autism spectrum disorder, and ADHD [18, 31, 57]. Our results herein agree with the previous studies indicating that UPF3B targets less transcripts for decay than core NMD factors such as UPF1, UPF2, SMG1, and SMG6 [9, 20, 21, 44, 45, 50]. It has been assumed that this is because UPF3B targets only a subset of transcripts detected by core NMD factors. However, to our knowledge, this has never been directly tested. Here, we report the surprising discovery that UPF3B and UPF2 drive the downregulation of largely different NMD-target mRNAs. As with the cell type-specific NMD-target mRNAs, we used stringent criteria, including pUPF1 occupancy, to define these NMD targets (Figs 4 and 8). These NMD targets are likely to be physiologically relevant, as we defined them in primary cells, not immortalized or malignant cell lines.

A major question in the field is how UPF3B acts in the NMD pathway. One possibility is that UPF3B is a “NMD amplifier” that quantitatively increases the magnitude of NMD. This possibility is consistent with the results of past studies [9, 44, 45, 55], but, to our knowledge, has not been directly tested. We addressed this issue by examining a set of mRNAs that we found depend on both UPF3B and the core NMD factor UPF2 for their decay by NMD. We found that these NMD targets are more strongly upregulated by UPF3B-KO coupled with UPF2 depletion than by UPF2 depletion alone—in both ESCs (Fig. 3E) and NPCs (Fig. 8C). This provides evidence that one function of UPF3B is to enhance NMD magnitude.

Another possibility is that UPF3B acts not only “quantitatively”, but also “qualitatively”, on the NMD pathway. Our study herein provided major support for this possibility, as we found that UPF3B drives the decay of mostly different mRNAs than does the core NMD factor UPF2 (Figs 4 and 8). How might UPF3B drive the decay of a unique set of NMD-target mRNAs? In addition to its UPF1- and EJC-interaction domains, UPF3B has a recently described “mid-domain” that supports NMD [44]. It will be interesting to determine whether this UPF3B mid-domain drives the decay of a unique set of NMD targets and, if so, how it accomplishes this molecularly. A non-mutually exclusive possibility is that UPF3B’s target specificity comes from UPF3B’s ability to drive translation termination, an event required for NMD [3]. UPF3B has been found to directly function in translation termination, at least *in vitro* [120], and thus this function might promote the decay of unique set of NMD-target transcripts. Heterogenous NMD responses may result from different classes of translation termination events; e.g., there is evidence that aberrant translation termination can elicit some forms of NMD [121].

By contrast with UPF3B, UPF2 is considered to be a core NMD factor, based on two lines of evidence. First, UPF2 provides several biochemical functions critical to the NMD pathway (see the “Introduction” section). Second, UPF2 is necessary for the decay of a large number of NMD targets. In agreement, our study identified 1706 and 1269 UPF2-dependent NMD-target mRNAs in NPCs and ESCs, respectively. We note that this may be an underestimate, as we did not completely eliminate UPF2 in our KO and KD experiments. By contrast, using the same approaches coupled with a complete UPF3B KO, we identified far fewer UPF3B-dependent NMD-target mRNAs: 503 and 391 in NPCs and ESC, respectively. Also consistent with UPF2 acting as a broadly acting NMD factor, it has been reported that loss of only one allele of *UPF2* upregulates hundreds of genes in lymphoblastoid cells [32]. While these findings support the notion that UPF2 is a core NMD factor required for the entire NMD pathway, it is possible that, like UPF3B, UPF2 drives the decay of only a subset of the entire spectrum of NMD-target mRNAs. This is supported by a seminal study that used RNAi to knock down UPF2 and some other NMD factors, followed by qPCR analysis of a small number of NMD-target mRNAs [122]. This study identified transcripts that were downregulated by NMD in HeLa cells even when UPF2 was knocked down, suggesting these transcripts are UPF2-independent NMD targets. In our study herein, we used genome-wide approaches to address the same question in primary cells. Our analyses identified many mRNAs degraded by NMD independently of UPF2 KO and KD in human ESCs and NPCs (Figs 2 and 5). However, it is important to note that, while we used several approaches to deplete UPF2, we did not completely eliminate UPF2, and thus we cannot rule out that some or all of the “UPF2-independent” target RNAs we defined require low levels of UPF2 to be degraded by NMD. If indeed there is a UPF2-dependent branch of NMD, this provides an explanation for why we found only modest overlap between UPF2- and UPF3B-dependent targets. Regardless of UPF2’s specificity, it will be fascinating to determine—at the molecular level—how UPF2 and UPF3B drive different transcripts to undergo rapid decay.

A complication of interpreting our findings from UPF3B-KO ESCs and NPCs is the fact that UPF3B has a paralog called “UPF3A” that can partially replace the function of UPF3B if UPF3B is eliminated or knocked down in level [9, 44, 45], a finding we confirmed (Fig. 4H). While interesting, we regard this “back up” function as unlikely to be UPF3A’s normal role, as this function would presumably only come into play during the occasional instances when UPF3B function is lost; e.g., when the *UPF3B* gene undergoes somatic mutation in an individual. Given that the *UPF3A* gene has been maintained in all vertebrates (i.e. for ∼500 million years), it has likely acquired specialized roles apart from UPF3B. In support, conditional KO studies indicate that UPF3A, not UPF3B, promotes spermatocyte progression in mice [55]. This function of UPF3A is consistent with the fact that its X-linked paralog, UPF3B, is absent from spermatocytes as a result of meiotic sex chromosome inactivation, a transcriptional silencing mechanism operating specifically in spermatocytes [55]. Given that UPF3A has been shown by multiple studies to be a weak NMD factor [123], this may explain why UPF3A has evolved to be expressed at extremely high levels in spermatocytes [55]. UPF3A may also have non-NMD functions; e.g., studies in zebrafish have shown that UPF3A functions in the “transcriptional adaptation” response that upregulates the paralogs of genes harbouring frame-disrupting mutations [124,125]. Just as UPF3A has likely acquired new functions since the *UPF3* gene duplication event, evidence suggests that UPF3B has also acquired specialized functions in vertebrates, particularly in the nervous system [18]. As evidence for this, *Upf3b*-KO mice suffer from behavioural and dendritic spine defects [21], and mouse neural stem and progenitor cells depleted of UPF3B suffer from defects in differentiation and neuronal maturation [51]. In humans, *UPF3B* mutations cause intellectual disability and are associated autism and schizophrenia [18, 31, 57]. Given that UPF3B has its own unique functions that appear to be separable from those of UPF3A, we feel it was justified to define NMD-target mRNAs that specifically require UPF3B, particularly since we defined these RNA targets in neural lineage cells—human NPCs. That said, it will be important for future investigations to also investigate RNAs that can be interchangeably degraded by NMD via UPF3A or UPF3B.

Our study also provides information on NMD-inducing features in different classes of mRNAs in normal cells. For example, we obtained strong correlative evidence that the most well-established NMD-inducing feature—an exon–exon junction >50-nt downstream of the main ORF [3–6, 40, 41] (i.e. what we refer to as a “dEJ”)—triggers both UPF2- and UPF3B-dependent NMD in both human ESCs and NPCs. However, our data also clearly demonstrate that the presence of a dEJ in an mRNA does not necessarily mean that the mRNA will be degraded by NMD. For example, we identified hundreds of dEJ-containing transcripts that are degraded in ESCs but not NPCs, as well as vice versa. We also obtained correlative evidence that another well-established NMD-inducing feature—a long 3′ UTR—commonly triggers UPF2-dependent NMD in ESCs. In particular, we found that (i) long 3′ UTRs are enriched in transcripts upregulated by two of the three approaches we used to disrupt UPF2 in ESCs and (ii) the average length of the 3′ UTR is longer in upregulated versus downregulated transcripts in response to all three approaches we used to disrupt UPF2 in ESCs. In contrast, we did not find that long 3′ UTRs are enriched in UPF2-dependent NMD targets in NPCs or UPF3B-dependent NMD targets in either ESCs or NPCs. Indeed, we found that transcripts upregulated in NPCs in response to either UPF2 or UPF3B disruption have “shorter” average 3′ UTR length than those that are downregulated.

In conclusion, we provide evidence that NMD is not a rigid pathway that degrades specific sets of mRNAs, but rather a flexible pathway whose mRNA targets depend on cellular context and the presence or absence of specific protein factors. Through transcriptome-wide analysis, we found that the mRNAs degraded by NMD differ in different cell types; even in different regions of the same organ. We also found that the NMD pathway selects mRNAs to be degraded in a factor-specific manner different than previously recognized. These findings have implications for understanding how NMD evolved, how it functions, how its magnitude is measured, and its roles in disease.

## Supplementary Material

gkaf395_Supplemental_Files

## Data Availability

The RNA-seq and pUPF1 RIPseq datasets generated in this study have been deposited at NCBI’s GEO database under the accession numbers: GSE263400, GSE263401, GSE263402, GSE263403, GSE263404, GSE263405, and GSE263406. No unpublished custom code, software, or algorithm were used in this study.
