# Supplementary material for: Cell type- and factor-specific nonsense-mediated RNA decay
Source: Nucleic Acids Res. 2025 May 14;53(9):gkaf395. doi: 10.1093/nar/gkaf395 (PMC12076418; doi:10.1093/nar/gkaf395)
Supplement: gkaf395_Supplemental_Files [file gkaf395_supplemental_files.zip › Supplementary Figures_gkaf395_FINAL_MW_5-7-25.pdf]

Supplementary Figure S1

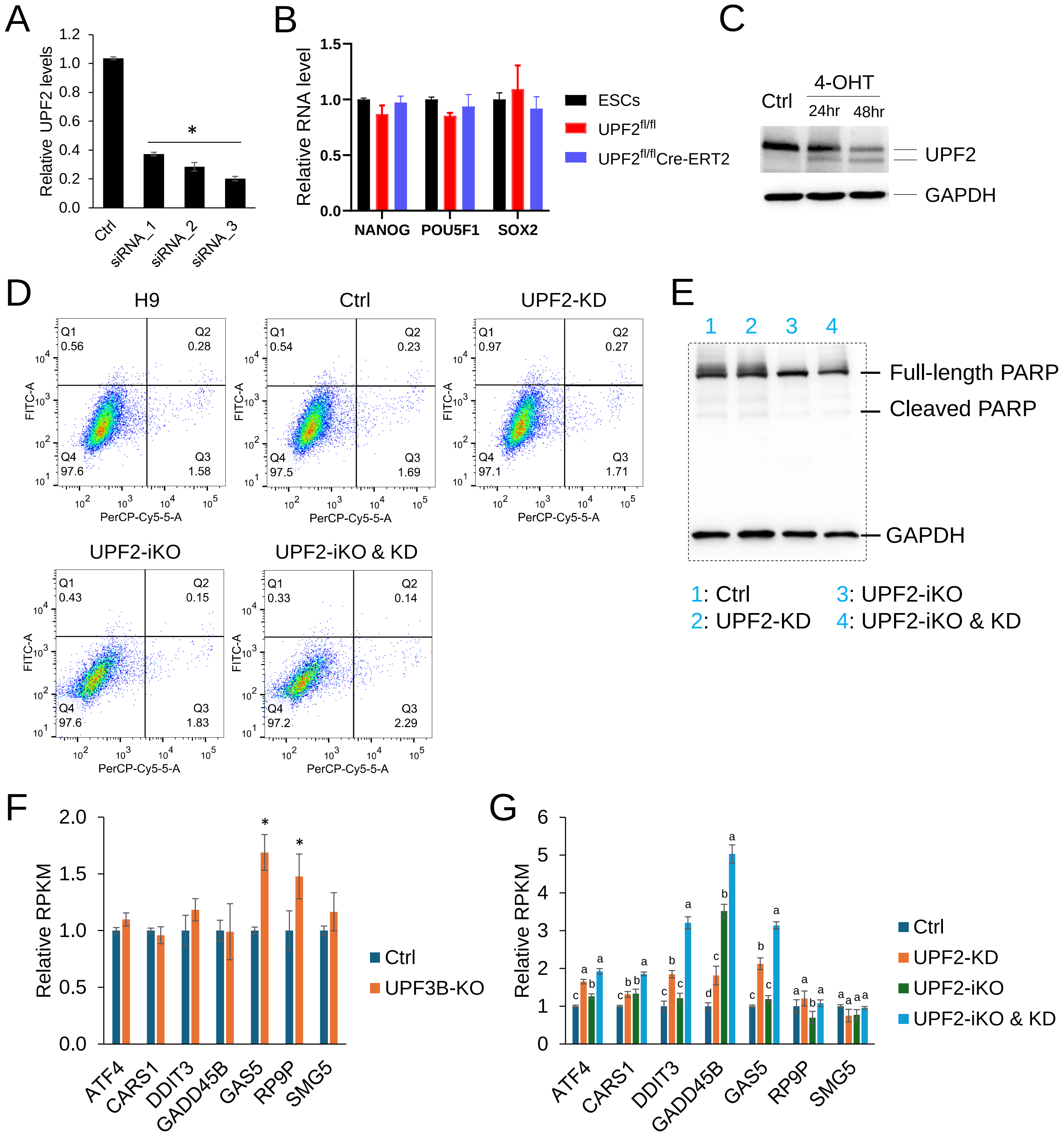

Supplementary Figure S1: UPF2-depleted and UPF3B-KO human ESCs. (**A**) qPCR analysis of ESCs transiently transfected with 3 different siRNAs targeting UPF2 or a negative-control (scrambled) siRNA (Ctrl). Statistical significance was determined using the Student's t test (n = 3). * p <0.05 as compared to the Ctrl. (**B**) qPCR analysis of the indicated pluripotency marker genes in ESC lines with the indicated genotype. The values shown for "$UPF2^{fl/fl}$" are from 2 independently-derived $UPF2^{fl/fl}$ ESC clones (2 biological replicates of each). The values shown for "$UPF2^{fl/fl}$ Cre-ERT2" are from 2 independently-derived $UPF2^{fl/fl}$ Cre-ERT2 ESC clones generated from the 2 $UPF2^{fl/fl}$ ESC clones described above (2 biological replicates for each). There is no statistically significant difference in expression of the 3 pluripotency genes, as determined using the Student's t-test. (**C**) Western blot analysis of UPF2-iKO ($UPF2^{fl/fl}$ Cre-ERT2) ESCs treated with 4-OHT for the indicated time periods. GAPDH is the loading control. Shown is a representative result from 2 independent experiments. (**D**) Apoptosis/necrosis analysis of ESCs cultured under the indicated conditions. H9, the parental cell line; Ctrl, $UPF2^{fl/fl}$ ESCs transiently transfected with a scramble siRNA; UPF2-KD, $UPF2^{fl/fl}$ ESCs transiently transfected with siUPF2; UPF2-iKO, $UPF2^{fl/fl}$ Cre-ERT2 ESCs transiently transfected with scramble siRNA; and UPF2-iKO & KD, $UPF2^{fl/fl}$ Cre-ERT2 ESCs transiently transfected with UPF2 siRNA. Both $UPF2^{fl/fl}$ and $UPF2^{fl/fl}$ Cre-ERT2 ESCs were incubated with 4-OHT for 48 hrs. All transfected cells were cultured for 48 hrs after transfection. Annexin V-FITC+ labels cells undergoing early apoptosis, while cells labeled by both Annexin V-FITC+ and PI+ are necrotic. (**E**) Western blot analysis of ESCs cultured under the indicated conditions, detailed in panel (D). GAPDH is the loading control. Shown is a representative result from 2 independent experiments. (**F** & **G**) Expression of known NMD-target RNAs in ESCs of the indicated genotypes, treated as indicated, based on RNA-seq analysis (see Figs 1 and 3). Expression in the control (Ctrl) ESCs was set as '1.' RPKM, reads per kilobase per million mapped reads. Different letters (a, b, c, and d) or * denote statistically significant differences between different groups (q <0.05).

Supplementary Figure S2

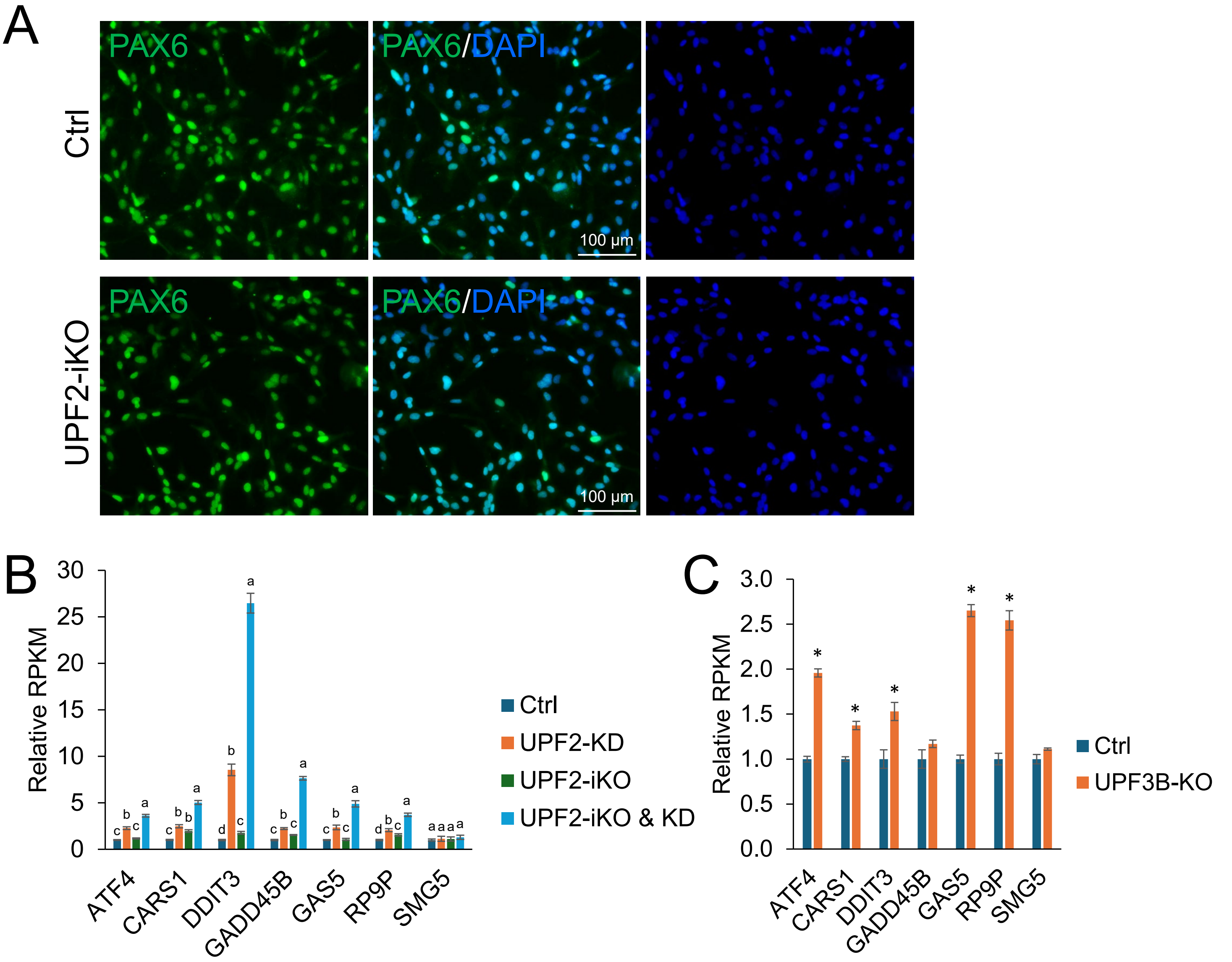

Supplementary Figure S2: Human NPCs derived from UPF2$^{fl/fl}$ and UPF2$^{fl/fl}$ Cre-ERT2 human ESCs. (**A**) Immunofluorescence analysis of NPCs generated from human ESCs with indicated genotypes. The results show that human NPCs derived from UPF2$^{fl/fl}$ (Ctrl) and UPF2$^{fl/fl}$ Cre-ERT2 (UPF2-iKO) ESCs express the NPC marker PAX6. The cells were also stained with DAPI (blue) to mark nuclei. Scale bar, 100 μm. (**B** & **C**) Expression of known NMD-target RNAs [8,47,54,104,106] in *UPF2*-depleted (**B**) and *UPF3B*-KO NPCs (**C**), based on RNA-seq analysis. Ctrl group was set as ‘1.’ RPKM, reads per kilobase per million mapped reads. Different letters (a, b, c, and d) denote statistically significant differences between different groups (* q <0.05).

Supplementary Figure S3

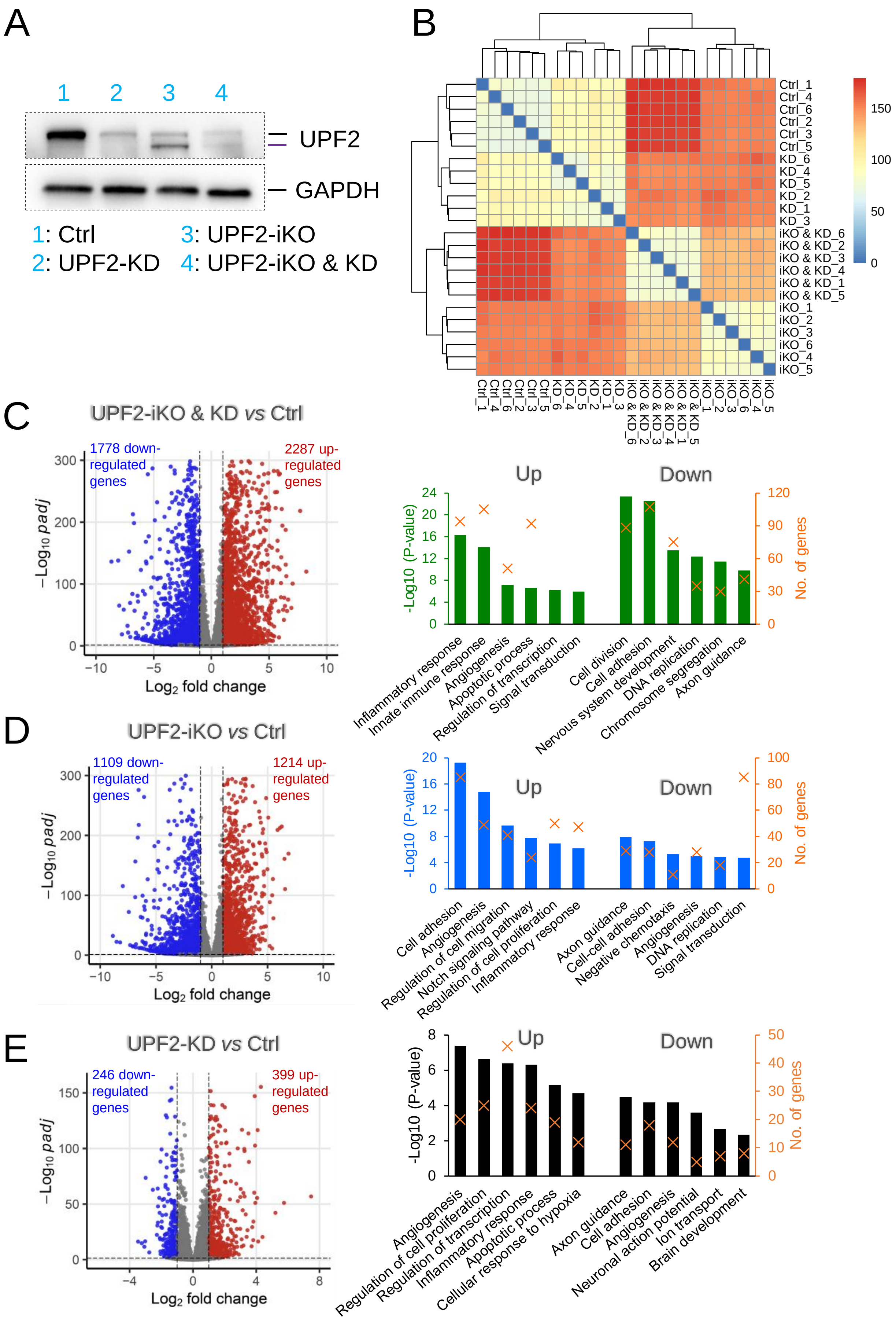

Supplementary Figure S3: Identification of UPF2-regulated genes in human NPCs. (**A**) Western blot analysis of NPCs cultured under the indicated conditions. Ctrl, $UPF2^{fl/fl}$ NPCs transiently transfected with a scramble siRNA; UPF2-KD, $UPF2^{fl/fl}$ NPCs transiently transfected with siUPF2; UPF2-iKO, $UPF2^{fl/fl}$ Cre-ERT2 NPCs transiently transfected with scramble siRNA; and UPF2-iKO & KD, $UPF2^{fl/fl}$ Cre-ERT2 NPCs transiently transfected with UPF2 siRNA. $UPF2^{fl/fl}$ and $UPF2^{fl/fl}$ Cre-ERT2 NPCs were incubated with 4-OHT for 48 hrs. All transfected cells were cultured for 48 hrs after transfection. UPF2-iKO generates a truncated version of UPF2. GAPDH is the loading control. n = 2. (**B**) Unsupervised hierarchical clustering of 2 independent clones of the $UPF2^{fl/fl}$ (Ctrl) and $UPF2^{fl/fl}$ Cre-ERT2 (UPF2-iKO) NPCs analyzed by RNA-seq (3 biological replicates performed per sample). UPF2 iKO and knockdown (KD) were performed as in Fig 1B. (**C-E**) Left, DEGs (q <0.01, fold change >2) identified from RNA-seq analysis. Right, biological functions associated with the DEGs defined in the panel to the left. Statistical significance [-Log10 (P-value)] is indicated by the bar. The number of DEGs for a given category is indicated by an "X."

Supplementary Figure S4

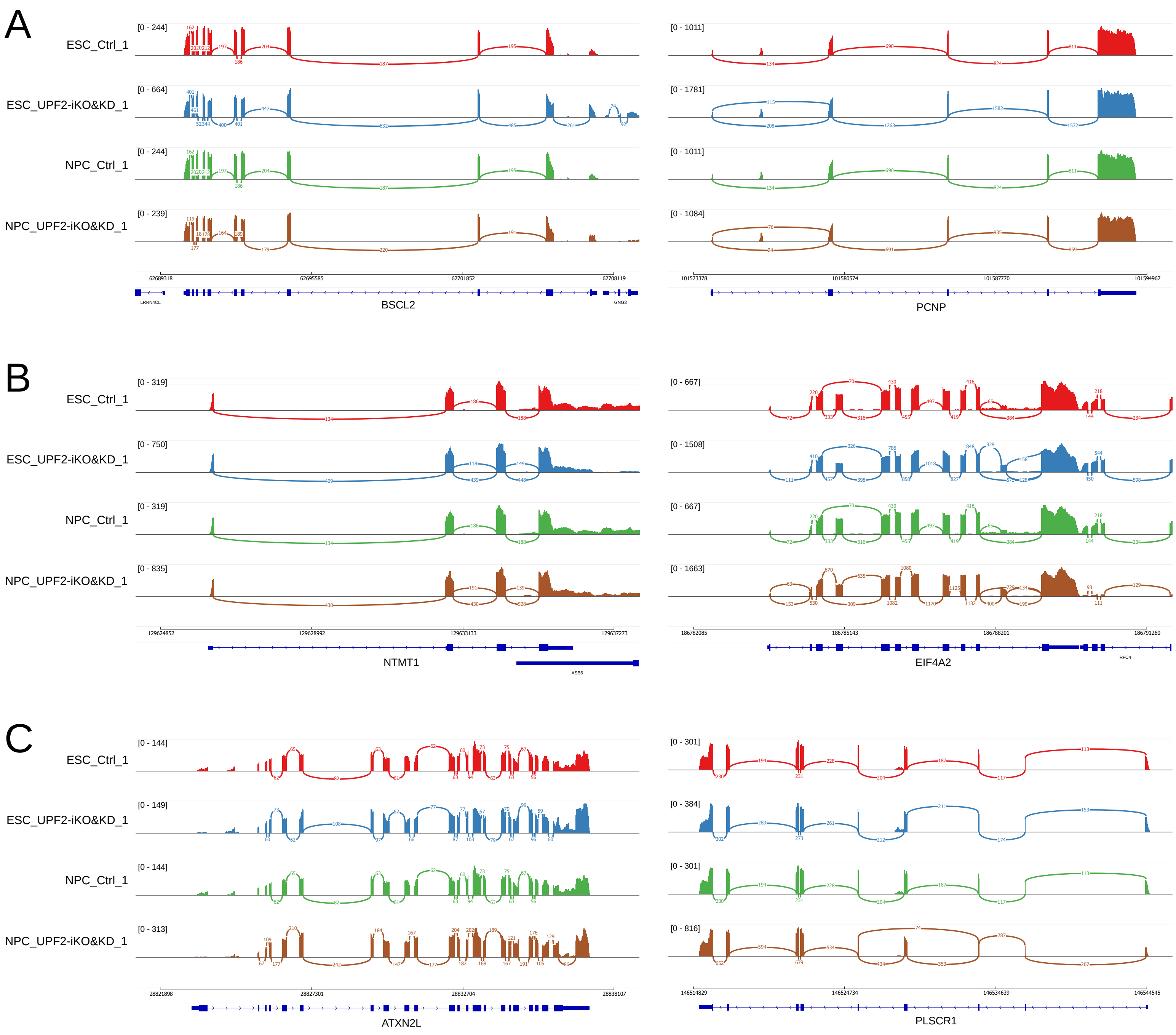

Supplementary Figure S4: Different classes of alternatively-spliced NMD-target RNAs. Sashimi plots showing representative ESC-specific (**A**), common (**B**), and NPC-specific (**C**) NMD-target transcripts. The data plotted is from RNA-seq analysis of UPF2-iKO & KD *vs.* control (Ctrl) cells described in Fig 2. Exon usage is indicated by the numbers associated with the curved lines linking read peaks. To save space, only replicate #1 is shown for each group.

Supplementary Figure S5

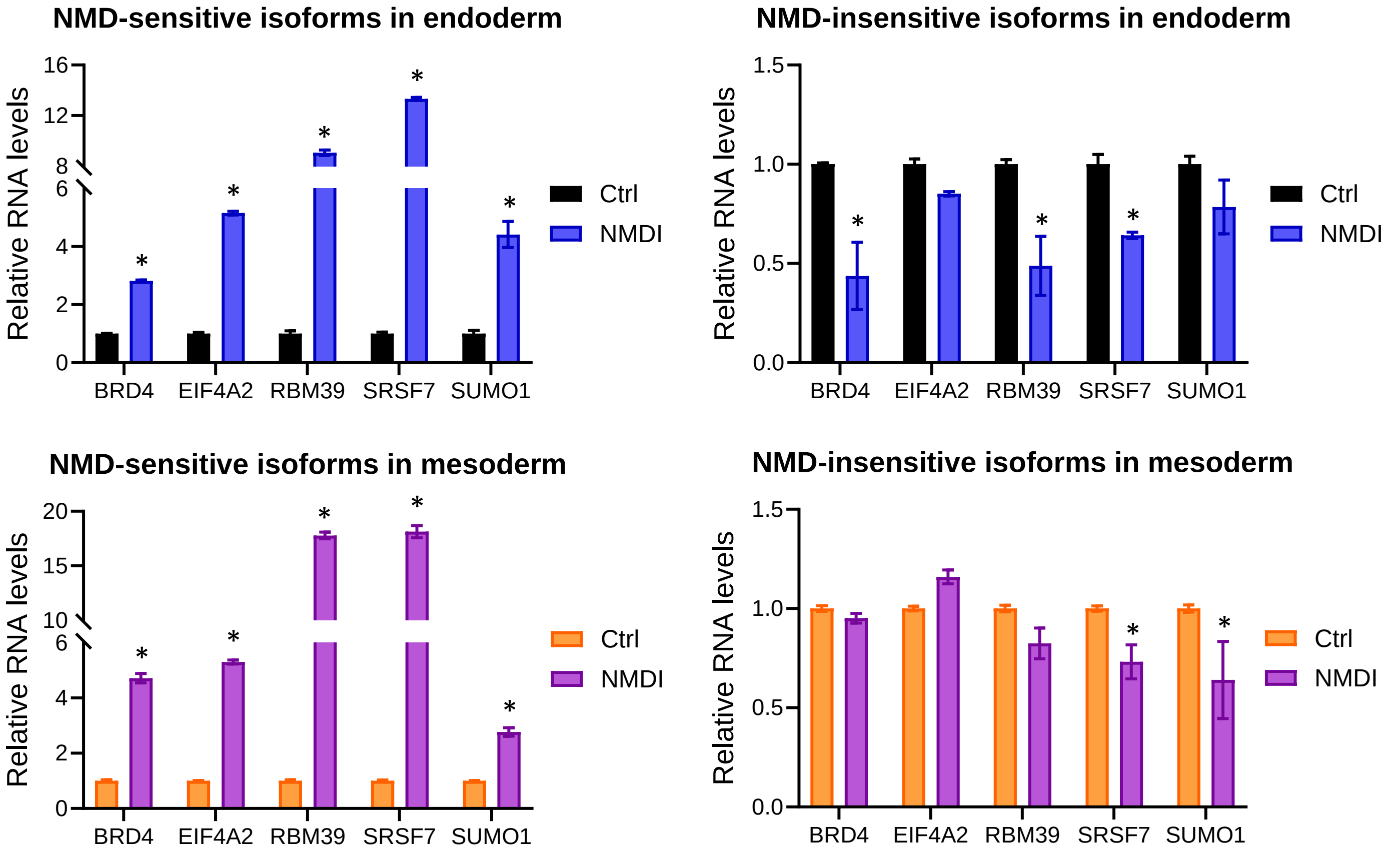

Supplementary Figure S5: NMD reporter transcripts. qPCR analysis of NMD-target mRNAs in human ESC-derived endoderm and mesoderm treated with the small-molecule NMD inhibitor, NMDI [113] or the vehicle, DMSO, alone (Ctrl). The NMD-sensitive transcripts (left) were defined as NMD targets in both ESCs and NPCs (Supplementary Table S5). The NMD-insensitive mRNA isoforms expressed from the same genes (right) control for potential changes in transcription. Together with the data in Supplementary Table S5, the data show that these 5 transcripts are NMD reporters in human ESCs, NPCs, mesoderm, and endoderm. Statistical significance was determined using the Student's t test. * p <0.05 (n=3).

Supplementary Figure S6

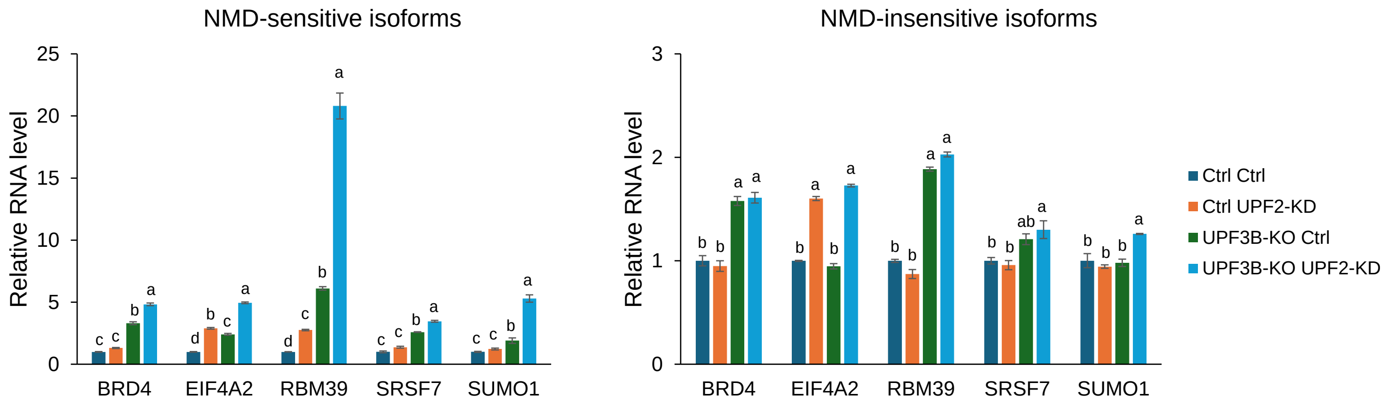

Supplementary Figure S6: UPF2 and UPF3B act either additively or synergistically on NMD reporter mRNAs in human NPCs. qPCR analysis of the NMD-sensitive and insensitive mRNA isoforms (see Fig 6H) in NPC lines (control [Ctrl] and *UPF3B*-KO) transiently transfected with scramble or UPF2 siRNAs. The values shown are from 2 independently-derived *UPF3B*-KO clones, with two independent biological replicates. Statistical significance was determined using the Student's t test. Different letters (a, b, c, and d) denote statistically significant differences between different groups ($p < 0.05$).
